# Chromium Catalysts for Selective Ethylene Oligomerization Featuring Binuclear PNP Ligands

**DOI:** 10.3390/molecules29092158

**Published:** 2024-05-06

**Authors:** Xiangyang Meng, Zhiqiang Ding, Huan Gao, Zhe Ma, Li Pan, Bin Wang, Yuesheng Li

**Affiliations:** Tianjin Key Laboratory of Composite & Functional Materials, School of Materials Science and Engineering, Tianjin University, Tianjin 300350, China

**Keywords:** PNP ligands, ethylene oligomerization, 1-octene

## Abstract

A series of novel binuclear PNP ligands based on the cyclohexyldiamine scaffold were synthesized for this study. The experimental results showed that positioning the two PNP sites at the para-positions of the cyclohexyl framework led to a significant enhancement in the catalytic activity for selective tri/tetramerization of ethylene. The PNP/Cr(acac)_3_/MAO(methylaluminoxane) catalytic system exhibited relatively high catalytic activity (up to 3887.7 kg·g^−1^·h^−1^) in selective ethylene oligomerization with a total selectivity of 84.5% for 1-hexene and 1-octene at 40 °C and 50 bar. The relationship between the ligand structure and ethylene oligomerization performance was further explored using density functional theory calculations.

## 1. Introduction

Linear alpha-olefins are important raw materials that can enhance the properties of polyolefins [1,2,3]. Introducing 1-hexene and 1-octene to high-density polyethylene (HDPE) or linear low-density polyethylene (LLDPE) significantly increases the branching of PE [4,5]. As a result, the copolymer has decreased crystallinity, a decreased melting point, and decreased density, which leads to exceptional flexibility and tear resistance. LLDPEs that contain comonomers 1-hexene or 1-octene exhibit higher tensile strength, impact resistance, tear resistance, and durability compared to those using 1-butene as a comonomer. The use of this PE resin as a high-value additive can result in the creation of thin yet highly flexible and robust LLDPE films [6,7,8,9,10,11,12,13]. Additionally, 1-hexene and 1-octene are utilized in the production of polyolefin elastomers (POE) [14]. Although there are numerous procedures for the production of 1-butene, with many functional applications, only a few manufacturers possess the technology required to produce 1-hexene and 1-octene, resulting in relatively limited domestic production capacity.

**Scheme 1:**
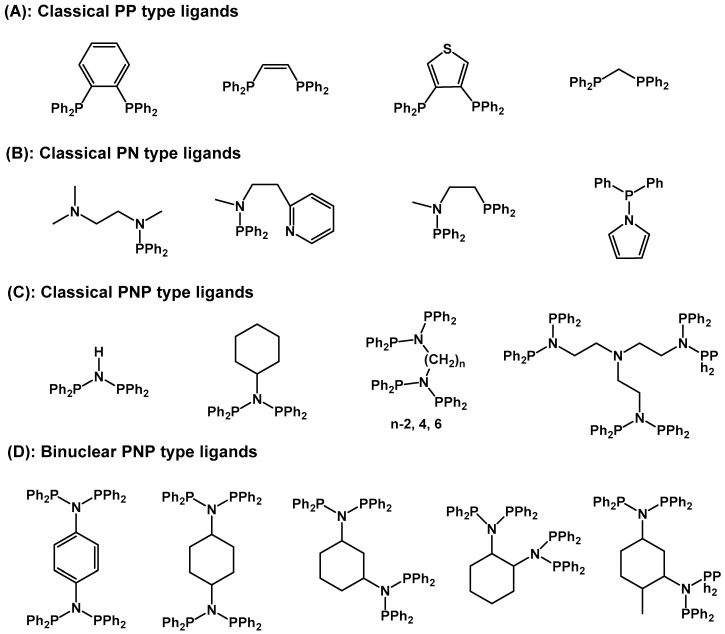
(**A**) Classical PP-type ligands used for ethylene oligomerization; (**B**) Classical PN-type ligands used for ethylene oligomerization; (**C**) Classical PNP-type ligands used for ethylene oligomerization; (**D**) Binuclear PNP-type ligands based on in this work.

Ethylene-selective oligomerization is an effective method for synthesizing 1-hexene and 1-octene. Phillips was the first company to industrialize ethylene trimerization in 2003, achieving 93% selectivity for 1-hexene [15,16]. In 2014, Sasol successfully produced ethylene tetramerization on an industrial scale [17,18]. The search for effective new catalysts has intensified due to the rapid development of selective ethylene polymerization. Currently, catalytic systems for the selective trimerization and tetramerization of ethylene are mainly based on chromium-based catalysts with phosphine amine ligands. As shown in Scheme 1, there are three primary types of ligands: PP, PN, and PNP ligands. 

The PP-type ligands include PNNP, PCnP, and PCNCP [19,20,21,22,23]. In 2004, Bolm and colleagues from Sasol reported a PNNP backbone ligand. The PNNP/Cr(acac)_3_/MAO catalytic system exhibited an activity of 26.2 kg/(g·h) at 45 °C and 45 bar, with a selectivity of 57.9% for 1-octene [19]. In 2008, Sasol researchers, Overett and colleagues, reported a series of PCnP backbone ligands. The most active system achieved a productivity of 2240 kg/(g·h), with the content of 1-octene reaching up to 62.3% [20]. Overett and colleagues also discovered that modifying the substituents could significantly alter the catalytic performance. In 2009, Floch and colleagues reported that a PCNCP/CrCl_3_(THF)_3_/MAO system could efficiently catalyze the selective trimerization of ethylene, with a maximum activity and selectivity for 1-hexene of 5.3 kg/(g·h) and 99%, respectively [23]. They also found that catalytic activities are higher when P is substituted with alkyl groups rather than phenyl groups. The catalytic performance of the system with cyclohexyl-substituted ligands was superior. Replacing the cyclohexyl group with a smaller tert-butyl group on the P atom favored ethylene tetramerization. However, the overall activity of this catalytic system remains relatively low.

PN ligands are frequently synthesized to selectively trimerize or tetramerize ethylene. In 2012, Gambarotta et al. reported three new phosphine amine ligands, Ph_2_PN(Me)(CH)_2_-X [X = NMe_2_, Py, PPh_2_], which demonstrated high selectivity in the trimerization/tetramerization of ethylene when mixed with trivalent metal sources and DMAO [24]. Sydora and colleagues reported on a chromium complex with an NCNP backbone ligand that can selectively catalyze the trimerization and tetramerization of ethylene with high selectivity when activated using MMAO-3A [25]. The catalytic activity increased gradually and reached to 1052 kg/(g·h), with the substitution of the phenyl group on the P atom with ethyl and then isopropyl. In 2015, Dyer and colleagues reported on a PCN backbone ligand. They found that ligands substituted with alkyl groups on P exhibited higher catalytic activities and produced less PE compared to those substituted with aryl groups [26].

Tridentate heteroatom bisphosphine PNP-type ligands are commonly used for selective ethylene polymerization thanks to their strong coordination ability and potential for industrialization. The substituents on the P and N atoms can be varied in PNP-chromium catalytic systems. These ligands are more widely applied compared to PP- and PN-type ligands owing to their ease of synthesis and the high activity of resulting chromium-based catalysts.

The studies have examined different structures of PNP ligands, such as N-substituted PNP ligands and P-substituted PNP ligands. N-substituted PNP ligands can be classified into three types: N-Alkyl-substituted PNP ligands, N-Cyclohexyl-substituted PNP ligands, and N-Aryl-substituted PNP ligands [19,27,28,29,30,31]. P-substituted PNP ligands can be categorized into four main types, including P-Aryl-substituted PNP ligands, PNPO ligands derived from PNP, asymmetric P-substituted PNP ligands, and phenyl interlinked PNP ligands [32,33,34,35,36,37,38]. Binuclear and trinuclear PNP ligands are also being researched, but there are currently limited reports on them [27,30,39,40]. In 2006, Jiang and colleagues reported a series of binuclear PNP ligands bridged by alkyl groups. They found that the activity gradually increased as the length of the alkyl bridge increased, while the selectivity remained unchanged [39].

The ratio of 1-hexene to 1-octene in linear alpha-olefins is related to the steric hindrance and electronic effects of substituents on the nitrogen atom of the PNP ligands. To investigate the relationship between ethylene copolymerization and ligand steric hindrance, we synthesized several binuclear PNP ligands with a cyclohexane framework. The PNP/Cr(acac)_3_/MAO catalytic system exhibited relatively high catalytic activity (up to 3887.7 kg·g^−1^·h^−1^) in ethylene-selective oligomerization with a total selectivity of 84.5% for 1-hexene and 1-octene at 40 °C and 50 bar. The relationship between the ligand structure and the ethylene oligomerization performance was further explored using density functional theory calculations [41].

## 2. Results and Discussion

### 2.1. Optimization of Ligands

**Figure 1:**
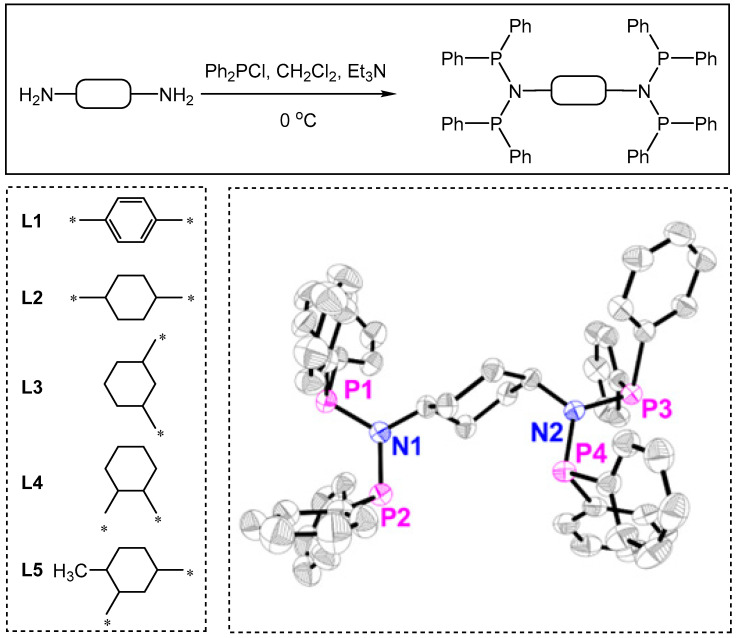
Synthesis of binuclear PNP-type ligands and the molecular structure of **L2**.

**Figure 2:**
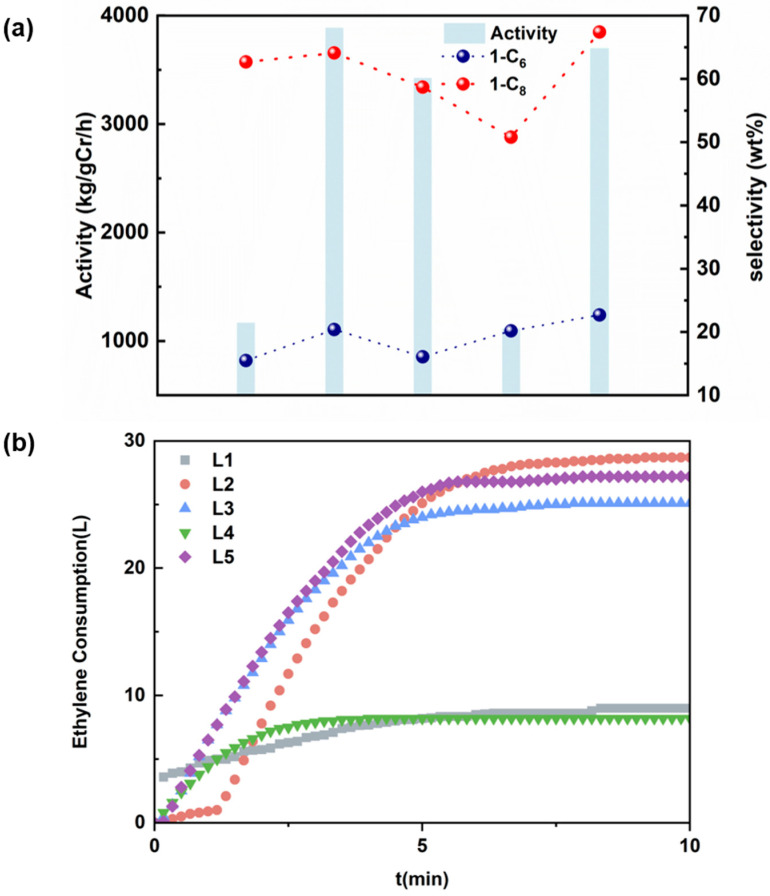
(**a**) The impact of ligand structure on catalytic activity and selectivity. (**b**) Ethylene consumption curves of PNP ligands 1–5.

The binuclear PNP-type ligands were synthesized from the reaction of diamine with Ph_2_PCl in the presence of Et_3_N. The structure of **L2** was confirmed using X-ray diffraction (Figure 1). Crystal data and structure refinement of **L2** were demonstrated in Supplementary Materials Table S1). All the ligands were identified via ^1^H, ^31^P, and ^13^C NMR and high-resolution MS (Figures S1–S20, Supplementary Materials). To evaluate the catalytic performance of the synthesized ligands, we conducted ethylene-selective oligomerization at 50 bar and 40 °C. The typical results are presented in Table 1. Because of the conjugation effect and steric hindrance of the benzene ring, the stability of the active center derived from **L1** and the metal source was relatively low. However, when the skeleton changed from a benzene ring to cyclohexyldiamine, both catalytic activity and selectivity of **L2** showed significant improvement. The catalytic system composed of **L2**/Cr(acac)_3_/MAO showed a selectivity of 64.1% for 1-octene in the liquid-phase products, with a total selectivity of 84.5% for 1-hexene and 1-octene (Figure S21 and Table S2, Supplementary Materials). The activity reached a high value of 3887.7 kg/(g·h). Figure 2a illustrates the catalytic properties of the five ligands. The five ligands exhibited different properties, which were related to the electronic and steric effects of the ligands. **L5** exhibited higher catalytic activity and selectivity than **L3**. The selectivity for 1-octene was 67.4% (Figure S22 and Table S3, Supplementary Materials), suggesting a potential stabilizing impact of the methyl group at the para-position on the active center, which improves catalytic activity and selectivity.

Figure 2b displays the ethylene consumption curves for the five ligands under the conditions of 40 °C, 50 bar, an Al/Cr ratio of 1000, and methylcyclohexane as the solvent. Ethylene consumption was measured using an ethylene gas mass flow meter. The rate curves for **L1**–**L5** exhibit typical decaying kinetics, characterized by a very high initial rate followed by rapid attenuation. It is important to note that **L2** exhibited the slowest decay rate, while **L4** exhibited the fastest decay rate and became deactivated in approximately 5 min. Based on these findings, **L2** demonstrated superior performance. As a result, future experiments will primarily investigate the **L2**-derived catalytic system.

The catalytic system composed of **L2**/Cr(acac)_3_/MAO (Table 1, entries 6–8) shows different effects with various solvents. We conducted a systematic investigation of the impact of methylcyclohexane (entry 6), cyclohexane (entry 7), and xylene (entry 8) on the catalytic performance of the **L2**/Cr(acac)_3_/MAO system in the selective polymerization of ethylene. Table 1 shows that **L2** exhibited higher catalytic activity when methylcyclohexane was used as the solvent. The improved solubility of ethylene gas in methylcyclohexane leads to a significant increase in the concentration of ethylene molecules around the catalyst’s active sites.

### 2.2. The Impact of Temperature on Ethylene Oligomerization

**Figure 3:**
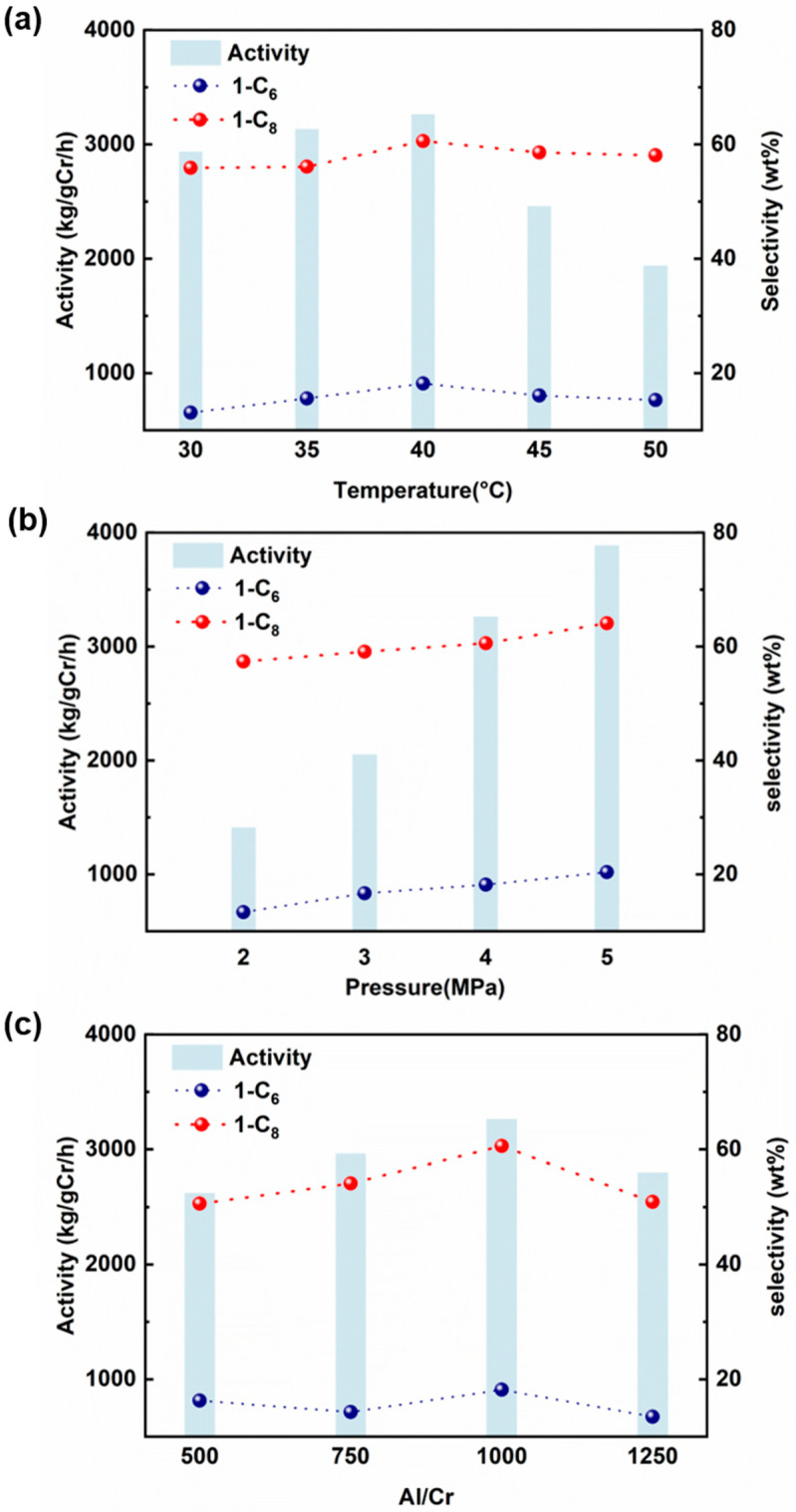
(**a**) The impact of temperature on catalytic activity and selectivity. (**b**) The impact of ethylene pressure on catalytic activity and selectivity. (**c**) The impact of the Al/Cr molar ratios on catalytic activity and selectivity.

The effect of reaction temperature on the catalytic performance of the **L2**/Cr(acac)_3_/MAO catalytic system was further investigated under the conditions of an Al/Cr molar ratio of 1000, reaction pressure of 40 bar, and reaction time of 10 min (Table 2 and Figure 3). The results indicate that catalytic activity and selectivity for 1-octene initially increased with the rise in reaction temperature but then decreased. At a reaction temperature of 40 °C, the catalytic activity reached its maximum at 3262.4 [kg/(g·h)], with a selectivity of 60.6% for 1-octene (entries 3). Lower reaction temperatures may not provide sufficient heat for the ethylene polymerization process, resulting in inadequate activation of the catalytic active centers. At higher reaction temperatures, the solubility of ethylene gas in the solvent decreases, leading to a reduced concentration of ethylene monomers in the solvent. Excessively high temperatures can lead to catalyst deactivation, which can cause a decrease in catalytic activity.

### 2.3. The Impact of Ethylene Pressures on Ethylene Oligomerization

The effects of different ethylene pressures on the ethylene-selective polymerization performance of the **L2**/Cr(acac)_3_/MAO catalytic system are summarized in Table 2 (entries 6–8) and Figure 3b. The table shows the results of our investigation on the influence of ethylene pressures ranging from 20 bar to 50 bar on the ethylene-selective polymerization reaction in the **L2**/Cr(acac)_3_/MAO catalytic system under the conditions of a reactor temperature of 50 °C, solvent methylcyclohexane, and an Al/Cr molar ratio of 1000. It has been shown that as the ethylene pressure increases, the solubility of ethylene gas in methylcyclohexane also increases. This leads to a higher concentration of ethylene molecules around the active centers, resulting in a gradual improvement in catalytic activity and selectivity for 1-octene.

### 2.4. The Impact of Al/Cr Molar Ratios on Ethylene Oligomerization

MAO plays a crucial role in ethylene oligomerization reactions by removing harmful impurities from the polymerization system, alkylating metal complexes, and reactivating inactive metal complexes formed by hydrogen transfer reactions. Therefore, the ratio of Al/Cr materials significantly affects the catalytic activity. A study was carried out to investigate the influence of different Al/Cr molar ratios on the performance of the **L2**/Cr(acac)_3_/MAO catalytic system in the selective polymerization of ethylene. The study was carried out under specific conditions, including an ethylene pressure of 40 bar, a temperature of 40 °C, and using methylcyclohexane as solvent. The results are shown in Table 3 (entries 1–4) and Figure 3c. Analysis of the graph shows that the activity of the catalyst increased as the Al/Cr molar ratio increased. However, when the Al/Cr molar ratio reached a certain value, the activity of the catalyst gradually decreased. The catalyst exhibited its highest activity at an Al/Cr molar ratio of 1000, which reached 3262.4 [kg/(g·h)]. A similar trend was observed in the selectivity for 1-octene, which peaked at 1000 with a selectivity of 60.6%. The results of the experiment indicate that the activity and selectivity of the **L2**/Cr(acac)_3_/MAO catalytic system are significantly influenced by the Al/Cr molar ratio. The observed trend is an overall increase followed by a decrease. One reason for this phenomenon is that air and other impurities present in the ethylene polymerization reactor may initially consume some cocatalyst. This results in a reduced amount of cocatalyst available to activate the main catalyst, resulting in an insufficient amount of the catalytic system participating in the reaction. However, if an excess of cocatalyst is added beyond the required amount, it may not only partially activate the main catalyst, but also potentially inhibit the progress of the reaction, thereby reducing the catalytic performance.

### 2.5. Relationship between Ligand Structure and Performance in Catalysts

**Figure 4:**
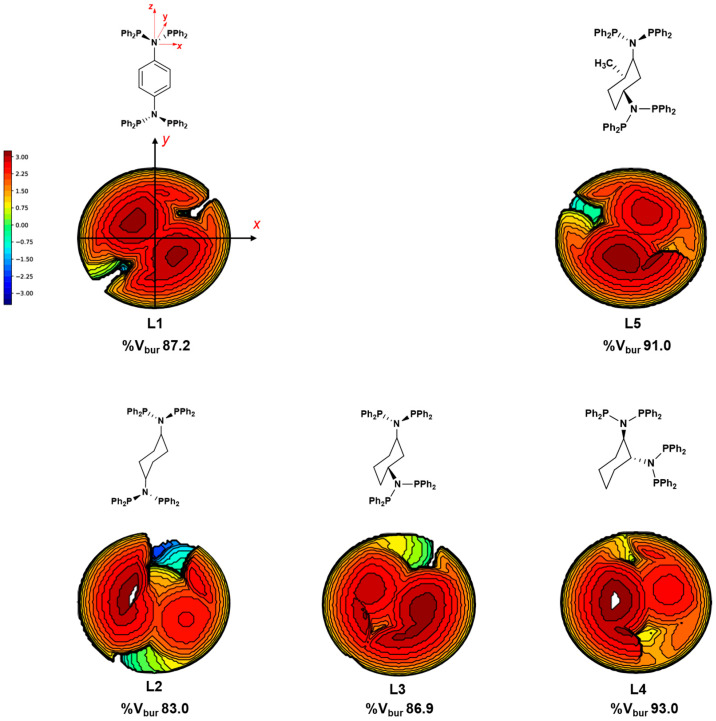
Steric maps of five ligands. The *z*-axis is defined by the nitrogen atom and its connected carbon atom, while the xz-plane is determined by the *z*-axis and a carbon atom on the phenyl ring.

To investigate the correlation between ligand steric hindrance and ethylene oligomerization performance, we optimized the structures of five ligands using density functional theory (DFT) and generated topographic steric maps for the five ligands, along with their respective buried volume percentages (%V_bur_) [42,43,44]. These maps illustrate the interaction surface between the catalyst and the substrate, with contour lines providing a quantitative description of the catalytic pocket. The buried volume percentage represents the degree of steric hindrance of the ligand (Figure 4).

**Figure 5:**
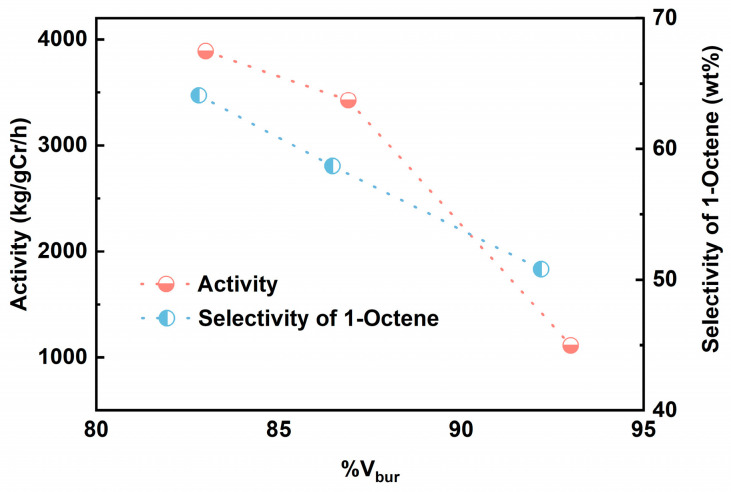
The trends in ethylene oligomerization activity and 1-octene selectivity with variations in the steric hindrance of the ligands 2–4.

The results are in agreement with previous findings. The %V_bur_ values for **L2**, **L3**, and **L4** are 83.0, 86.9, and 93.0, respectively. The gradual increase in %V_bur_ indicates that the spatial hindrance of the ligand increases as the two PNP active centers shift from the ortho- to the meta- and then to the para-position (refer to Figure 5). The activity of ethylene oligomerization and the selectivity for 1-octene gradually decrease with an increase in steric hindrance. This is because the ligand has a reduced ability to coordinate with the metal center, which is consistent with the findings reported by Killian [28].

When comparing **L1** with **L2**, an increase in steric hindrance results in a decrease in activity from 3887.7 [kg/(g·h)] to 1168.8 [kg/(g·h)], with %V_bur_ reaching 87.2. The decrease in activity is not only due to steric hindrance but is also related to electronic effects. The conjugation effect disperses the electron cloud density on PNP, reducing the ligand’s ability to coordinate with the metal center. When the two PNP active centers are connected via a benzene ring, this effect is more pronounced compared to cyclohexane. Additionally, the introduction of a methyl group resulted in increased steric hindrance for **L5** compared to **L3**, with %V_bur_ rising from 86.9 to 91.0. However, the activity increased from 3425.5 [kg/(g·h)] to 3697.4 [kg/(g·h)]. This could be ascribed to the fact that **L5** has better solubility in methylcyclohexane than **L3**. The introduction of a methyl group resulted in an increase in selectivity for 1-octene from 58.6% to 67.4%. This suggests that the methyl group aids in stabilizing the formation of a nine-membered ring during the ethylene coordination-insertion process, which favors the production of 1-octene [45,46,47].

**Figure 6:**
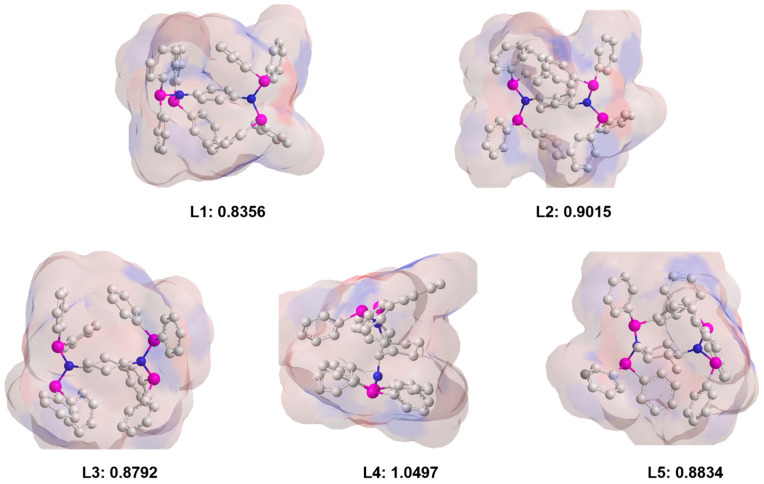
Charge density profiles of **L1**-**L5** and the average charge on the phosphorus atom.

To investigate the influence of electronic effects on **L1**–**L5**, we calculated the Huckel charges of five ligands and plotted their charge density distribution, as shown in Figure 6. The charge amount on the phosphorus atom is labeled in the figure. The results indicate that, compared to **L1**, **L2** shows a decrease in charge, which is unfavorable for coordination with chromium metal, resulting in lower catalytic activity. This is attributed to the dispersion of electron cloud density on the phosphorus atom due to the conjugation effect of the benzene ring. Among **L2**–**L4**, **L2** exhibits the highest charge amount on the phosphorus atom, reaching 1.0497. However, it demonstrates the lowest activity, suggesting that steric hindrance exerts a more significant influence on the ligand’s activity than electronic effects. Compared to **L3**, the ortho-methyl substituent in L5 increases the charge density on the phosphorus atom, leading to enhanced catalytic activity.

In addition, **L1** serves as a rigid ligand, maintaining its framework with minimal alteration throughout the coordination process. In contrast, **L2**–**L5** exhibit flexibility as ligands, enabling them to adopt diverse configurations in response to changes in the coordination environment. This flexibility allows them to display a degree of adaptability during coordination.

## 3. Materials and Methods

### 3.1. Materials

Unless otherwise stated, all chemicals are used without further purification. P-Phenylenediamine (99.9%), cyclohexane-1,4-diamine (98%), cyclohexane-1,3-diamine (95%), 1,2-Diaminocyclohexane (99%), Cr(acac)_3_ (98+%), anhydrous dichloromethane (99.9%), anhydrous tetrahydrofuran (99.9%), anhydrous cyclohexane (99.5%) and acetonitrile (99.9%) were purchased from Innochem (Beijing, China). Phenylphosphonic dichloride (PPh_2_Cl, 98%) and CrCl_3_(THF)_3_ (97%) were purchased from Macklin. Methylcyclohexane and xylene were obtained using the multi-column purification system. Both polymerization-grade ethylene (99.9%) and high-purity nitrogen (99.999%) were purchased from Tianjin Shengtang Gas Co., Ltd. (Tianjin, China). Methylaluminoxane (MAO, 1.5 mol/L in toluene) was purchased from Kagi Chemie.

### 3.2. Methods

^1^H, ^13^C, and ^31^P spectra were recorded on a Bruker Avance III spectrometer (400 MHz for ^1^H NMR, 101 MHz for ^13^C NMR, and 162 MHz for ^31^P NMR) at room temperature in chloroform-d (purchased form Innochem). A suitable crystal was selected and carried out on a Bruker D8 VENTURE dual wavelength Mo/Cu diffractometer [48,49,50]. The crystal was kept at 193.00 K during data collection. GC-MS spectra were recorded on a gas chromatography mass spectrometer by Agilent (7890A-5975C). All the density functional theory calculations were performed by Gaussian 09 program [51]. The GC-MC instrument worked at 50 °C for 3 min, and was then heated to 280 °C at 10 °C/min. The content of linear α-olefins was determined using the area normalization method and the solid product was dried for 12h in an oven at 50 °C to a constant weight.

### 3.3. Synthesis of Ligands

#### 3.3.1. *N*,*N*′-1,4-Phenylenebis [*N*-(diphenylphosphino)-*P*,*P*-diphenylphosphinous Amide](**L1**)

To prepare the compound, p-Phenylenediamine (1.08 g, 10 mmol) and triethylamine (4.55g, 45 mmol) were dissolved in 100 mL of dichloromethane. Ph_2_PCl (8.87 g, 40.2 mmol) was then slowly added at 0 °C. The mixture was stirred for 2 h at 0 °C and then allowed to warm up to room temperature and stirred for an additional 24 h. The volatiles were removed under reduced pressure, and the residue was extracted using anhydrous THF (3 × 10 mL). The solvent was removed, and the solid residue was mixed with anhydrous CH_3_CN (5 × 5 mL). The resulting compound was dried under vacuum to yield ligand 1 in a 70% yield. ^1^H NMR (400 MHz, CDCl_3_): δ 5.97 (s, 4 H), 7.14–7.42 (m, 40 H). ^13^C NMR (101 MHz, CDCl_3_): δ 127.91, 128.94, 129.25, 133.28, 139.08, and 142.92. ^31^P NMR (162 MHz, CDCl_3_): δ 70.27 (s).

#### 3.3.2. *N*,*N*′-1,4-Cyclohexyl [*N*-(diphenylphosphino)-*P*,*P*-diphenylphosphinous Amide](**L2**)

Cyclohexane-1,4-diamine (1.14 g, 10 mmol) and triethylamine (4.55 g, 45 mmol) were dissolved in 100 mL of dichloromethane. Ph_2_PCl (8.87 g, 40.2 mmol) was slowly added at 0 °C. The mixture was stirred for 2 h at room temperature and then for an additional 24 h. The volatiles were removed under reduced pressure, and the residue was extracted using anhydrous THF (3 × 10 mL). The solid residue was triturated with anhydrous CH_3_CN (5 × 5 mL) after removing the solvent. Ligand 2 was obtained in a yield of 63% after drying under vacuum. ^1^H NMR (400 MHz, CDCl_3_): δ 1.07 (m, 4 H), 2.10 (m, 4 H), 3.28 (m, 2 H), 7.14–7.23 (m, 40 H). ^13^C NMR (101 MHz, CDCl_3_): δ 30.70, 55.76, 127.95, 128.51, 132.91, and 140.24. ^31^P NMR (162 MHz, CDCl_3_): δ 51.69–52.67 (m).

#### 3.3.3. *N*,*N*′-1,3-Cyclohexyl [*N*-(diphenylphosphino)-*P*,*P*-diphenylphosphinous Amide](**L3**)

Cyclohexane-1,3-diamine (1.14 g, 10 mmol) and triethylamine (4.55 g, 45 mmol) were dissolved in 100 mL of dichloromethane. Ph_2_PCl (8.87 g, 40.2 mmol) was slowly added at 0 °C. The mixture was stirred for 2 h at 0 °C and then warmed to room temperature and stirred for an additional 24 h. The volatiles were removed under reduced pressure, and the residue was extracted using anhydrous THF (3 × 10 mL). The solvent was removed, and the solid residue was mixed with anhydrous CH_3_CN (5 × 5 mL). The resulting compound was dried under vacuum to yield ligand 3 in a 58% yield. ^1^H NMR (400 MHz, CDCl_3_): δ 0.44 (m, 1 H), 0.80 (d, 2 H), 1.18 (m, 1 H), 1.43 (m, 2 H), 2.07 (d, 2 H), 3.21 (m, 3 H), 7.11–7.21 (m, 40 H). ^13^C NMR (101 MHz, CDCl_3_): δ 22.86, 33.31, 43.11, 58.93, 127.95, 128.10, 133.64, and 139.92. ^31^P NMR (162 MHz, CDCl_3_): δ 51.24 (s).

#### 3.3.4. *N*,*N*′-1,2-Cyclohexyl [*N*-(diphenylphosphino)-*P*,*P*-diphenylphosphinous Amide](**L4**)

Ph_2_PCl (8.87 g, 40.2 mmol) was slowly added to a solution of cyclohexane-1,2-diamine (1.14 g, 10 mmol) and triethylamine (4.55 g, 45 mmol) in 100 mL of dichloromethane at 0 °C. The mixture was stirred for 2 h and then allowed to warm up to room temperature and stirred for an additional 24 h. The volatiles were removed under reduced pressure, and the residue was extracted using anhydrous THF (3 × 10 mL). The solvent was removed, and the solid residue was mixed with anhydrous CH_3_CN (5 × 5 mL). The resulting compound was dried under vacuum to yield ligand 4 in a 50% yield. ^1^H NMR (400 MHz, CDCl_3_): δ 87 (m, 2 H), 1.15 (m, 4 H), 2.47 (m, 2 H), 3.34 (m, 1 H), 3.72 (m, 1 H), 6.85–7.77 (m, 40 H). ^13^C NMR (101 MHz, CDCl_3_): δ 24.97, 25.67, 34.63, 58.05, 128.14, 131.81, 134.45, and 141.96. ^31^P NMR (162 MHz, CDCl_3_): δ 48.21 (s) and 58.28 (s).

#### 3.3.5. *N*,*N*′-1,3-Cyclohexyl [*N*-(diphenylphosphino)-*P*,*P*-diphenylphosphinous Amide](**L5**)

4-methylcyclohexane-1,3-diamine (1.28 g, 10 mmol) and triethylamine (4.55 g, 45 mmol) were dissolved in 100 mL of dichloromethane. Ph_2_PCl (8.87 g, 40.2 mmol) was then slowly added at 0 °C. The mixture was stirred for 2 h at 0 °C and then allowed to warm up to room temperature and stirred for an additional 24 h. The volatiles were removed under reduced pressure, and the residue was extracted using anhydrous THF (3 × 10 mL). The solvent was removed, and the solid residue was mixed with anhydrous CH_3_CN (5 × 5 mL). The resulting compound was dried under vacuum to yield ligand 5 in a 61% yield. ^1^H NMR (400 MHz, CDCl_3_): δ 0.23–2.95 (m 12 H),6.54–7.62 (m, 40 H). ^13^C NMR (101 MHz, CDCl_3_): δ 18.61, 32.66, 33.30, 35.44, 44.67, 58.86, 63.90, 127.24, 127.98, 128.40, 129.08, 129.59, 130.02, 130.93, 131.10, 131.84, 132.14, 132.69, 134.02, 134.24, 134.45, 135.00, 135.27, 136.24, 136.59, 139.58, and 139.87. ^31^P NMR (162 MHz, CDCl_3_): δ 48.38 (s), 49.43 (s), 51.76 (s), and 58.44 (s).

### 3.4. Ethylene Oligomerization

The oligomerization of ethylene was conducted in a 500 mL high-pressure, stainless-steel reactor with a magnetic stirring bar and temperature detection device. The reactor was vacuum-dried at 120 °C for 4 h and the ethylene gas was replaced three times. The reactor was then cooled to the desired temperature using circulating water cooling. The solvents, cocatalysts, ligands, and chromium sources were sequentially injected into the reactor under vacuum. The experiment began by introducing ethylene to the set pressure and activating the stirring and flow meters. Timing started at this point. After the reaction was complete, the reactor was cooled to room temperature, the pressure was released, and acidified ethanol (30 mL) was used to quench any oligomerization. The liquid phase was separated, and the product distribution was analyzed using gas chromatography mass spectrometry (GC-MS).

## 4. Conclusions

We synthesized and investigated a series of novel binuclear PNP ligands to explore the performance differences in ethylene oligomerization, where two PNP active centers are located at different positions (ortho-, meta-, and para-). Theoretical calculations and experimental results showed that the activity and selectivity for ethylene oligomerization decrease sequentially as the steric hindrance of the binuclear PNP ligands increases. The maximum catalytic activity for ethylene oligomerization is achieved when the two PNP active centers are in the ortho-position, demonstrating significant ethylene tetramerization activity. Ligand-2-based Cr catalyst shows great potential for further development due to its ability to maintain ethylene oligomerization performance for an extended period and exhibit high selectivity for linear α-olefins.

## Figures and Tables

**Table 1:** Ethylene tri-/tetramerization using Cr catalysts supported by ligands 1–5.

Entry *^a^*	Ligand	Activity [kg/(g·h)]	Product Selectivity (%)				
			1-C_6_ *^b^* (wt%)	1-C_8_ *^b^* (wt%)	(1-C_6_ + 1-C_8_) *^b^* (wt%)	C_10_^+^ *^b^* (wt%)	PE *^c^* (wt%)
1	**1**	1168.8	15.5	62.7	78.2	10.3	1.4
2	**2**	3887.7	20.4	64.1	84.5	9.8	1.7
3	**3**	3425.5	16.0	58.6	74.6	11.2	3.0
4	**4**	1114.7	20.2	50.8	71.0	13.9	10.1
5	**5**	3697.4	22.7	67.4	90.1	8.8	4.8
6 *^d^*	**2**	3262.4	18.2	60.6	78.8	9.6	1.5
7 *^e^*	**2**	2677.9	17.3	59.8	77.1	13.3	0.4
8 *^f^*	**2**	2215.9	16.9	56.1	76.0	14.8	0.2

*^a^* General conditions: 1.0 μmol of Cr(acac)_3_, 2 μmol of ligand, 1000 equiv. of MAO, 150 mL methylcyclohexane, 40 °C, 50 bar, run time: 10 min. *^b^* wt% of the liquid fraction. *^c^* wt% of the total product. *^d^* Pressure: 40 bar. *^e^* Pressure: 40 bar; Solvent: cyclohexane. *^f^* Pressure: 40 bar; Solvent: xylene.

**Table 2:** Ethylene tri-/tetramerization of ligand 2 under different temperatures and pressures.

Entry *^a^*	*T*/°C	P/bar	Activity [kg/(g·h)]	Product Selectivity (%)				
				1-C_6_ *^b^* (wt%)	1-C_8_ *^b^* (wt%)	(1-C_6_ + 1-C_8_) *^b^* (wt%)	C_10_^+^ *^b^* (wt%)	PE *^c^* (wt%)
1	30	40	2936.9	13.1	55.9	69.0	10.7	0.9
2	35	40	3134.1	15.6	56.1	71.1	10.3	1.1
3	40	40	3264.4	18.2	60.6	78.8	9.6	1.5
4	45	40	2460.4	16.1	58.6	74.7	12.3	1.2
5	50	40	1938.4	15.3	58.1	73.4	11.8	0.7
6	40	20	1413.7	13.4	57.4	70.8	13.4	0.4
7	40	30	2052.6	16.7	59.1	75.8	10.6	0.9
8	40	50	3887.7	20.4	64.1	84.5	6.8	1.7

*^a^* General conditions: 1.0 μmol of Cr(acac)_3_, 2 μmol of ligand, 1000 equiv. of MAO, 150 mL methylcyclohexane, run time: 10 min. *^b^* wt% of the liquid fraction. *^c^* wt% of the total product.

**Table 3:** Ethylene tri-/tetramerization of ligand 2 under different Al/Cr ratios.

Entry *^a^*	Al/Cr	Activity [kg/(g·h)]	Product Selectivity (%)				
			1-C_6_ *^b^* (wt%)	1-C_8_ *^b^* (wt%)	(1-C_6_ + 1-C_8_) *^b^* (wt%)	C_10_^+^ *^b^* (wt%)	PE *^c^* (wt%)
1	500	2623.5	16.3	50.6	66.9	15.7	1.4
2	750	2963.4	14.3	54.1	68.4	16.4	2.4
3	1000	3264.4	18.2	60.6	78.8	9.6	1.5
4	1250	2800.2	13.5	50.9	64.4	14.3	2.2

*^a^* General conditions: 1.0 μmol of Cr(acac)_3_, 2 μmol of ligand, 1000 equiv. of MAO, 150 mL methylcyclohexane, run time: 10 min. *^b^* wt% of the liquid fraction. *^c^* wt% of the total product.

## Data Availability

Data are contained within the article and Supplementary Materials.

## Supplementary Materials

The following supporting information can be downloaded at: https://www.mdpi.com/article/10.3390/molecules29092158/s1. Crystal data and structure refinement of L2 were demonstrated in Supplementary Materials (Table S1). Corresponding residence time of chromatographic peak to the product (Table S2). Corresponding residence time of chromatographic peak to the product (Table S3). All the ligands were identified via 1H, 31P, and 13C NMR and high-resolution MS (Figures S1–S20, Supplementary Materials). GC-MS spectrum of the oligomerization product obtained from entry 2 of Table 1 (Figure S21). GC-MS spectrum of the oligomerization product obtained from entry 5 of Table 1 (Figure S22).
