# Supplementary material for: Chromium Catalysts for Selective Ethylene Oligomerization Featuring Binuclear PNP Ligands
Source: Molecules. 2024 May 6;29(9):2158. doi: 10.3390/molecules29092158 (PMC11085890; doi:10.3390/molecules29092158)
Supplement: Supplementary file 1 [file molecules-29-02158-s001.zip › molecules-2947232-supplementary.pdf]

Electronic Supplementary Information

for

Chromium Catalysts for Selective Ethylene Oligomerization Featuring Binuclear PNP ligands

Xiangyang Meng, Zhiqiang Ding, Huan Gao, Zhe Ma, Li Pan, Bin Wang*, Yuesheng Li

Tianjin Key Laboratory of Composite & Functional Materials, School of Materials Science and Engineering, Tianjin University, Tianjin 300350, China.

CONTENTS

1. **Crystallographic details**
2. **Spectrum of Ligands**
3. **High Resolution Mass Spectrometry**
4. **GC-MS spectrum of the typical oligomerization products**
5. **Computational methodology**

1. Crystallographic details

A suitable crystal was selected and carried out on a Bruker D8 VENTURE dual wavelength Mo/Cu diffractometer. The crystal was kept at 193.00 K during data collection. Using Olex2 [1], the structure was solved with the SHELXT [2] structure solution program using Intrinsic Phasing and refined with the SHELXL [3] refinement package using Least Squares minimization.

Table S1. Crystal data and structure refinement of ligand **2**.

Crystal and structure data	Ligand **2**
Empirical formula	$C_{54}H_{50}N_2P_4$
Formula weight	850.84
Temperature [K]	193.00
Crystal system	orthorhombic
Space group (number)	$Pbca$ (61)
a [Å]	9.0993(10)
b [Å]	19.8172(17)
c [Å]	54.043(7)
α [°]	90
β [°]	90
γ [°]	90
Volume [Å^3]	9745.2(18)
Z	8
ρ_{calc} [gcm^{-3}]	1.160
μ [mm^{-1}]	1.704
F(000)	3584
2θ Range(°)	9.51 to 137.19 (0.83 Å)
Index ranges	$-10 \le h \le 10$
	$-17 \le k \le 23$
	$-65 \le l \le 62$
Reflections collected	53974
Goodness-of-fit on F2	1.035

Final R indices [I>2sigma(I)]	R_1 = 0.0957
Largest diff. peak and hol	0.28/−0.30

2. Spectrum of Ligands

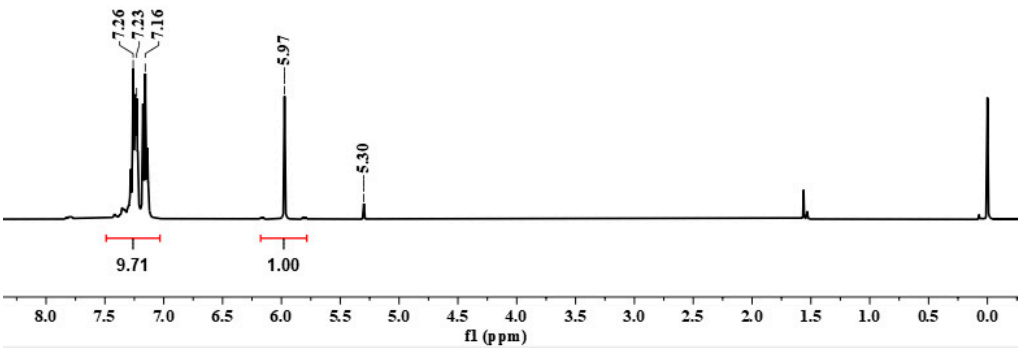

Figure S1. The ^{1}H NMR spectra of ligand **1**.

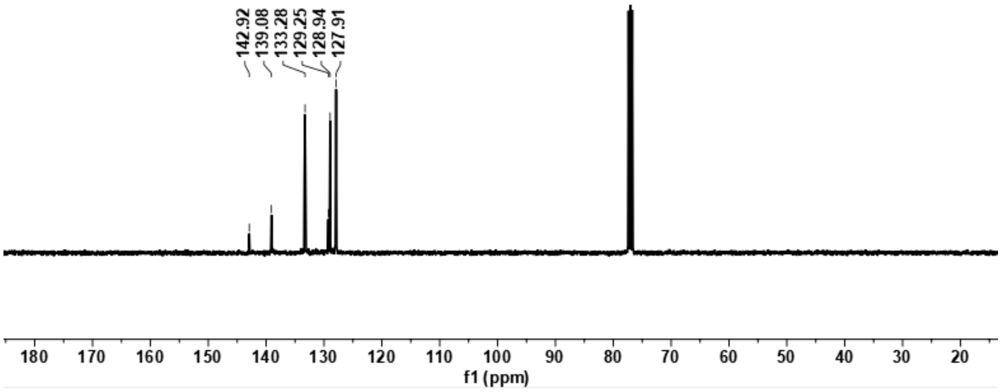

Figure S2. The ^{13}C NMR spectra of ligand **1**.

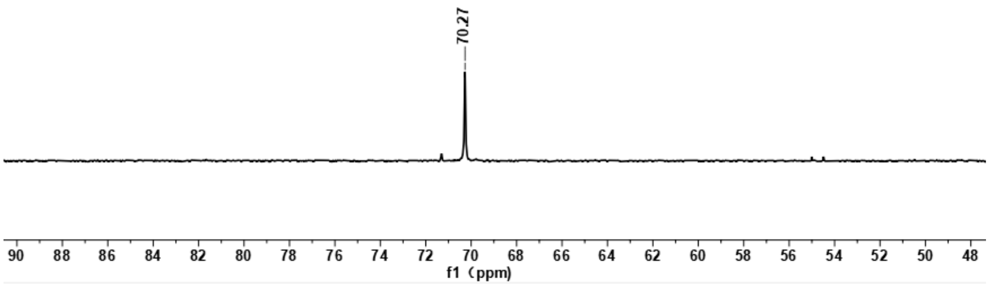

Figure S3. The ^{31}P NMR spectra of ligand **1**.

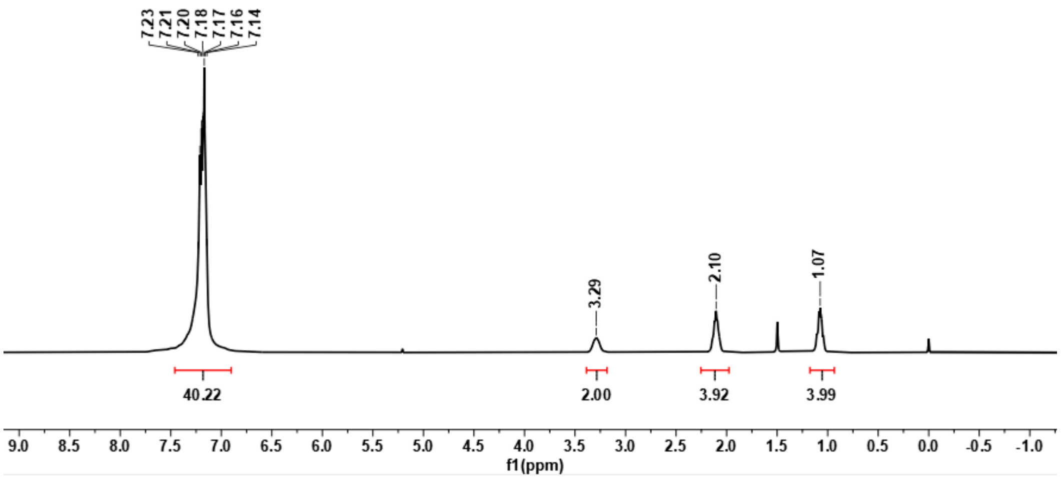

Figure S4. The ^{1}H NMR spectra of ligand **2**.

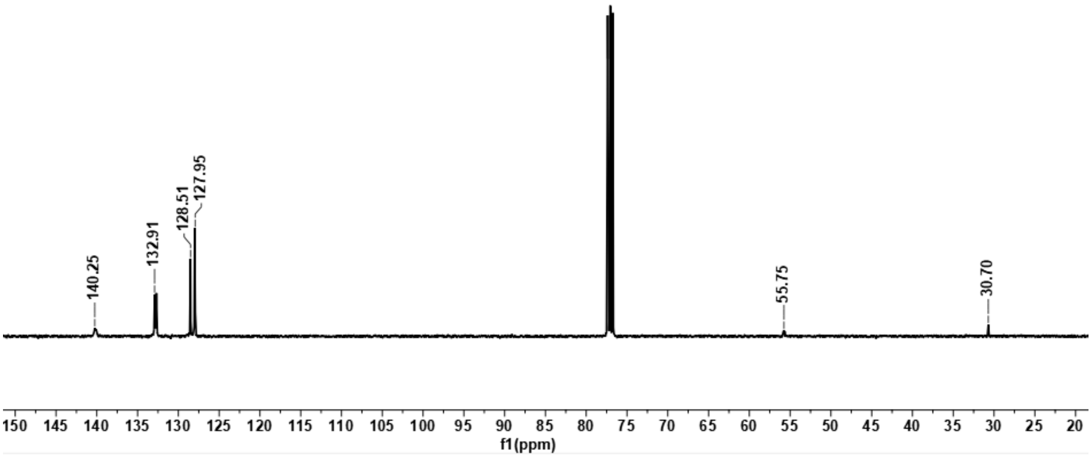

Figure S5. The ^{13}C NMR spectra of ligand **2**.

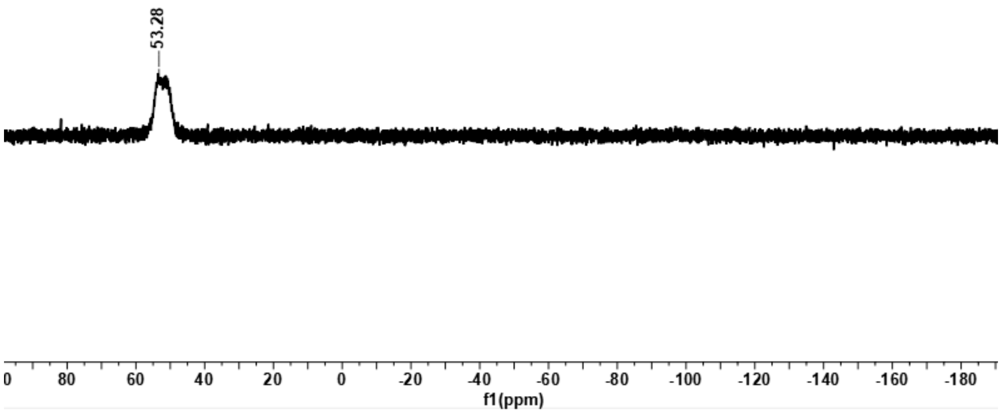

Figure S6. The ^{31}P NMR spectra of ligand **2**.

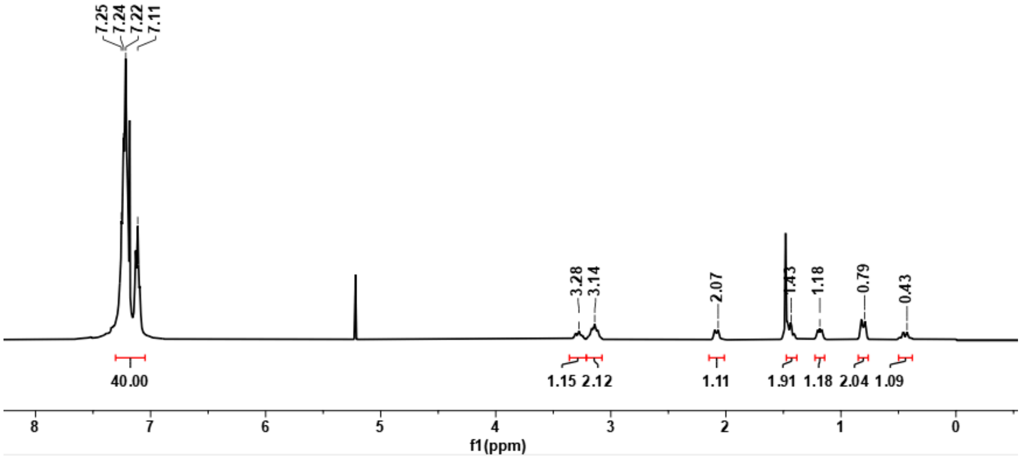

Figure S7. The ^{1}H NMR spectra of ligand **3**

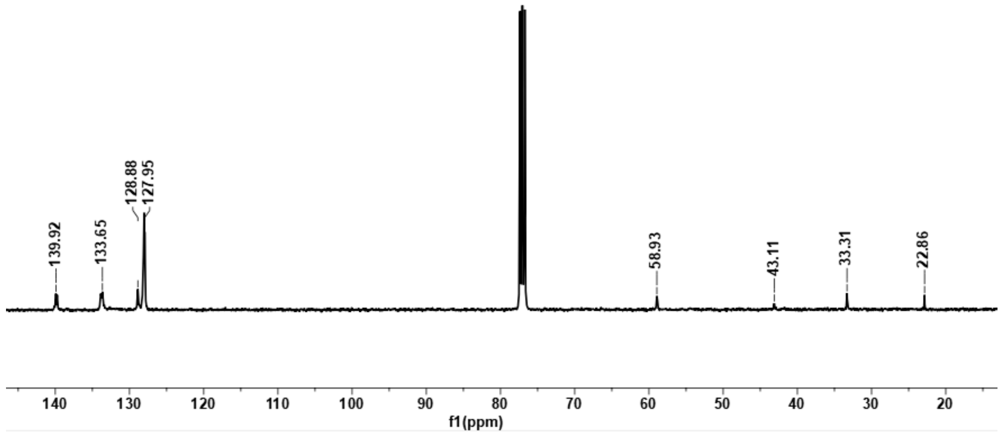

Figure S8. The ^{13}C NMR spectra of ligand **3**.

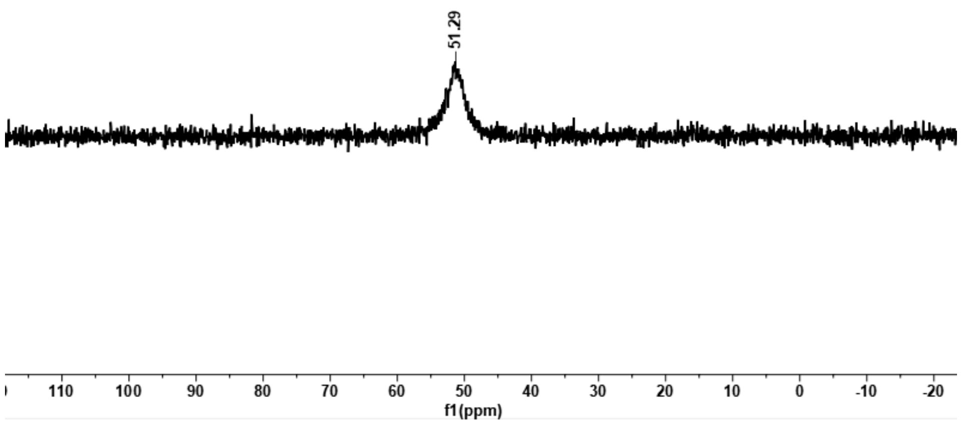

Figure S9. The ^{31}P NMR spectra of ligand **3**.

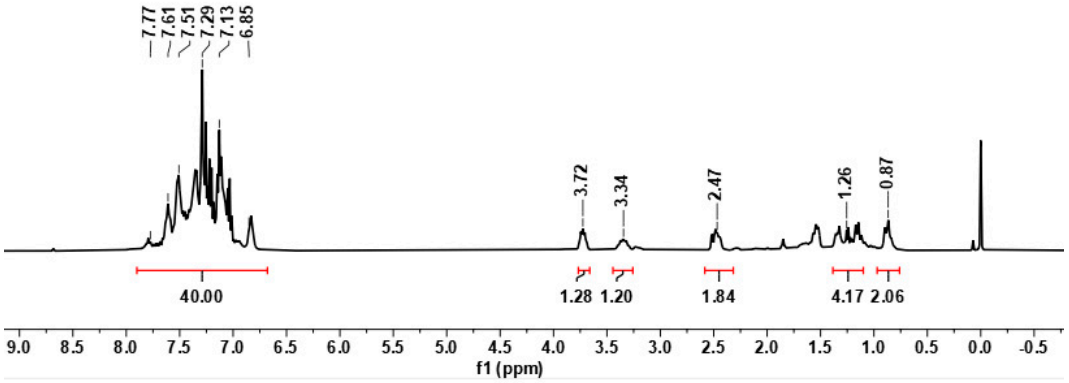

Figure S10. The ^{1}H NMR spectra of ligand **4**.

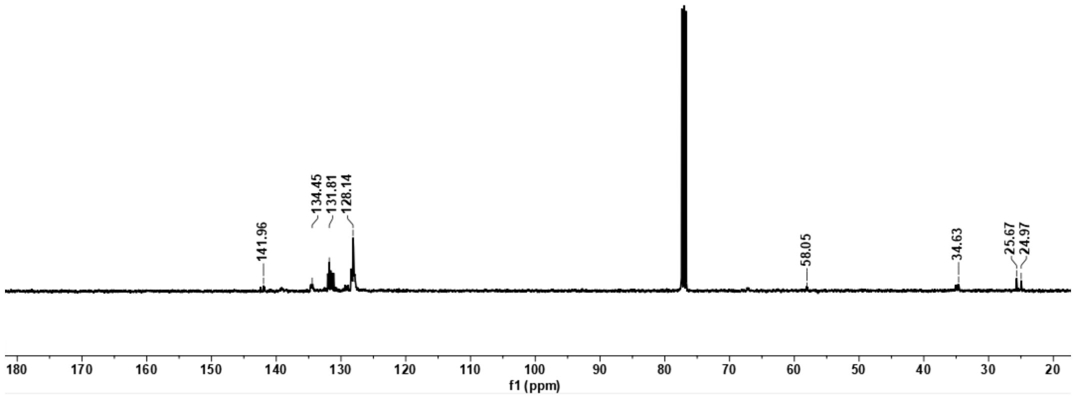

Figure S11. The ^{13}C NMR spectra of ligand **4**.

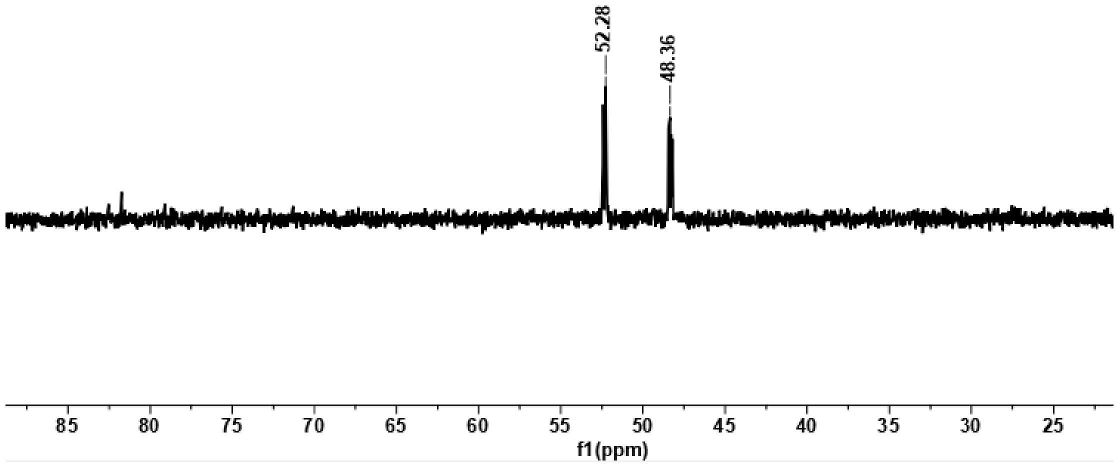

Figure S12. The ^{31}P NMR spectra of ligand **4**.

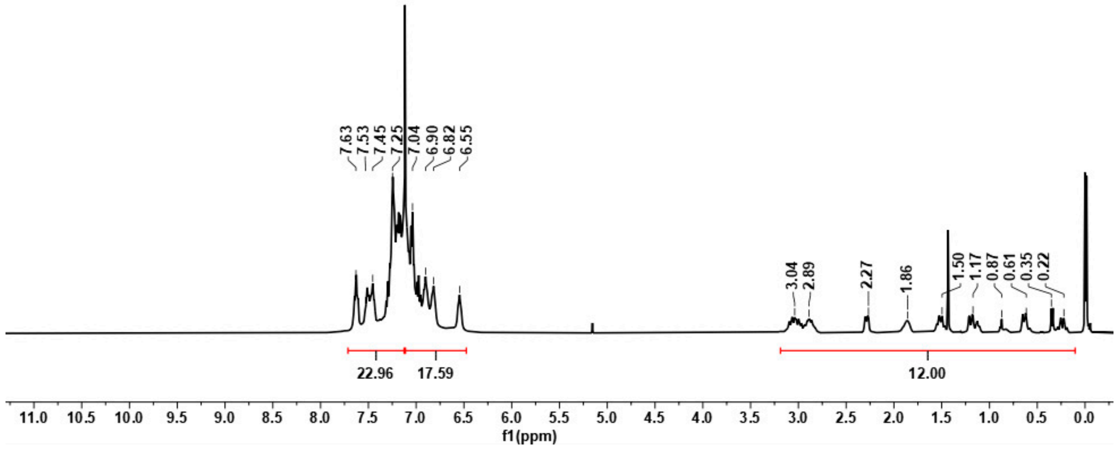

Figure S13. The ^{1}H NMR spectra of ligand **5**.

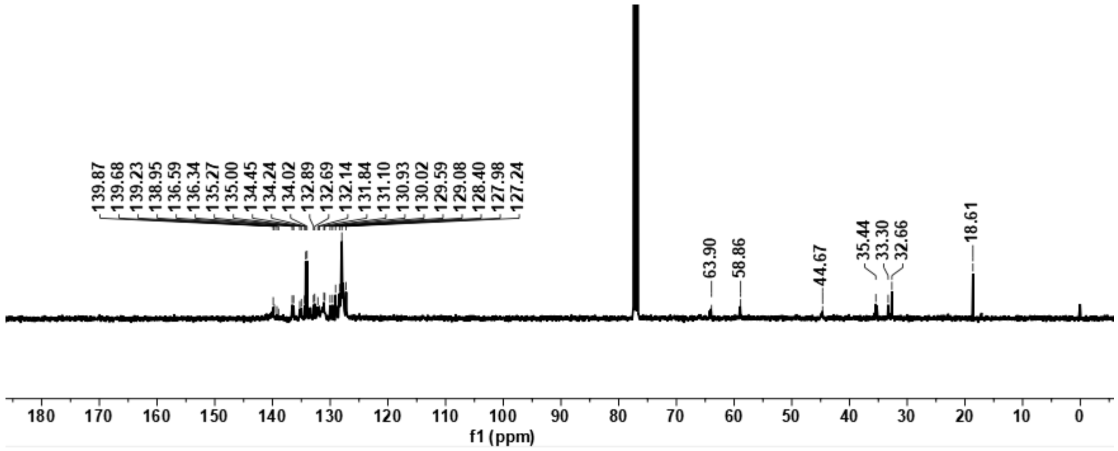

Figure S14. The ^{13}C NMR spectra of ligand **5**.

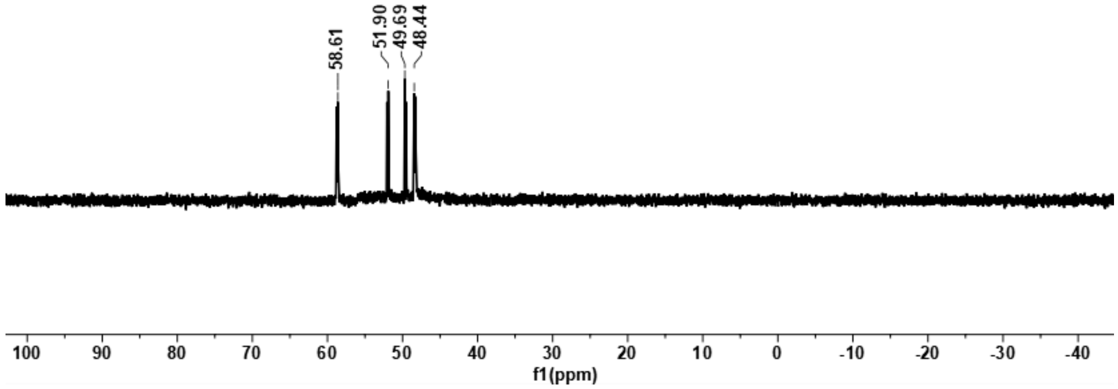

Figure S15. The ^{31}P NMR spectra of ligand **5**.

6. High Resolution Mass Spectrometry of Ligands

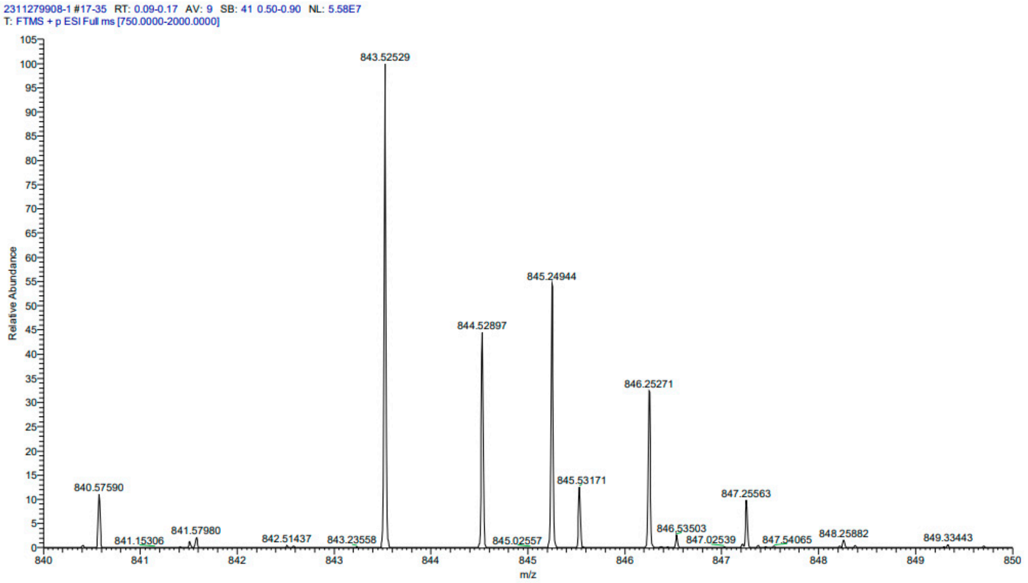

Figure S16. High Resolution Mass Spectrometry of **L1**

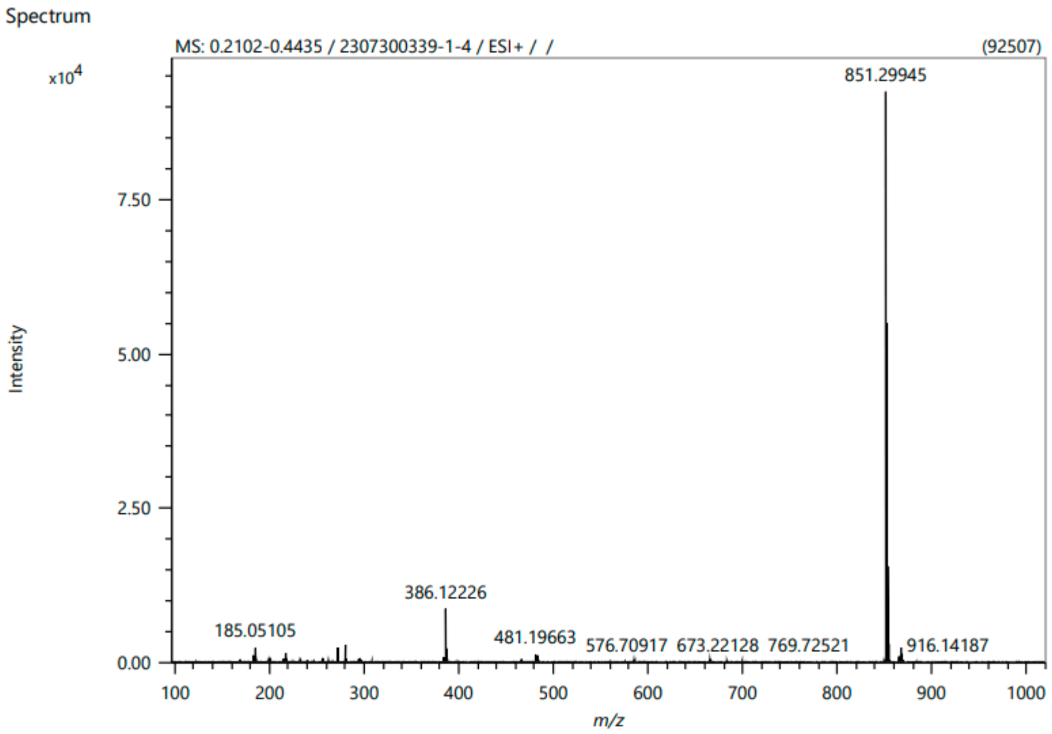

Figure S17. High Resolution Mass Spectrometry of **L2**

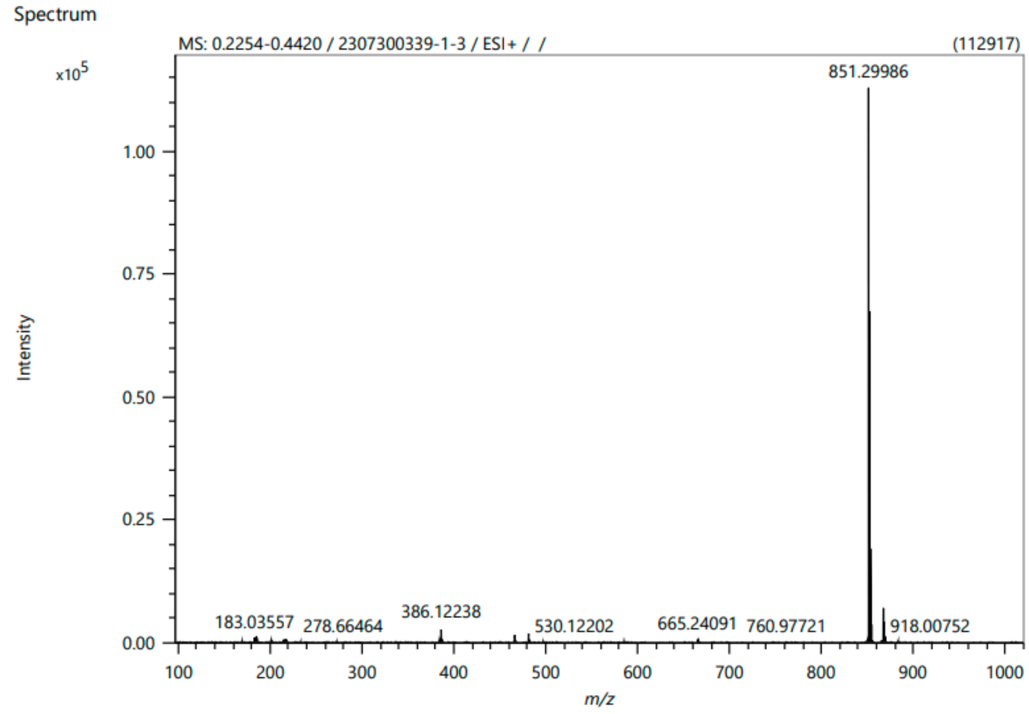

Figure S18. High Resolution Mass Spectrometry of **L3**

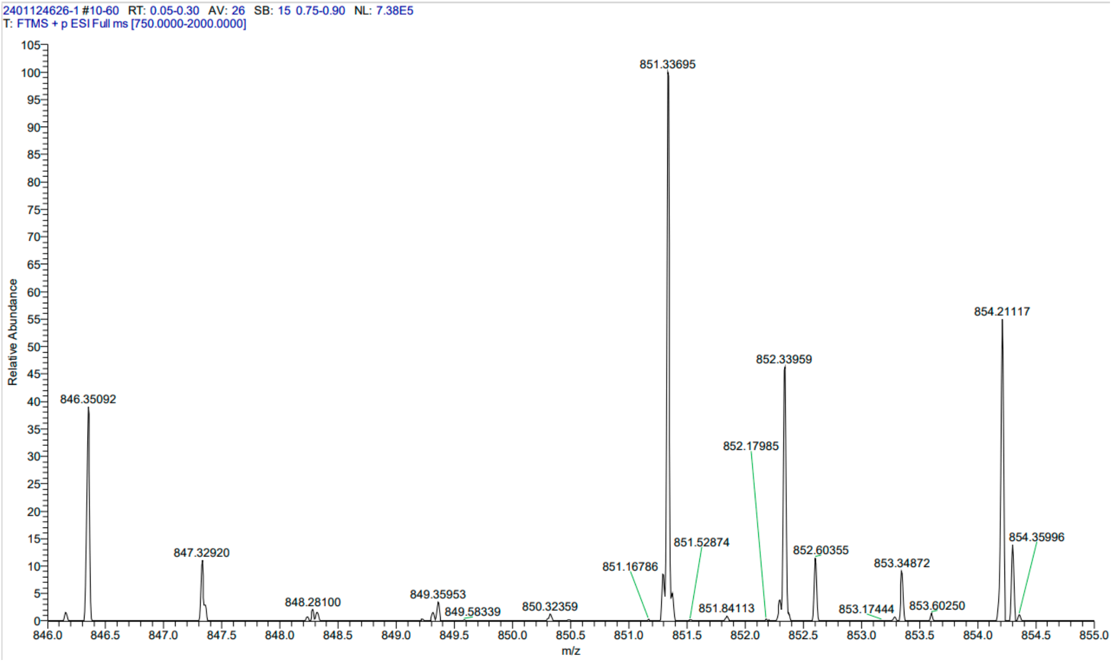

Figure S19. High Resolution Mass Spectrometry of **L4**

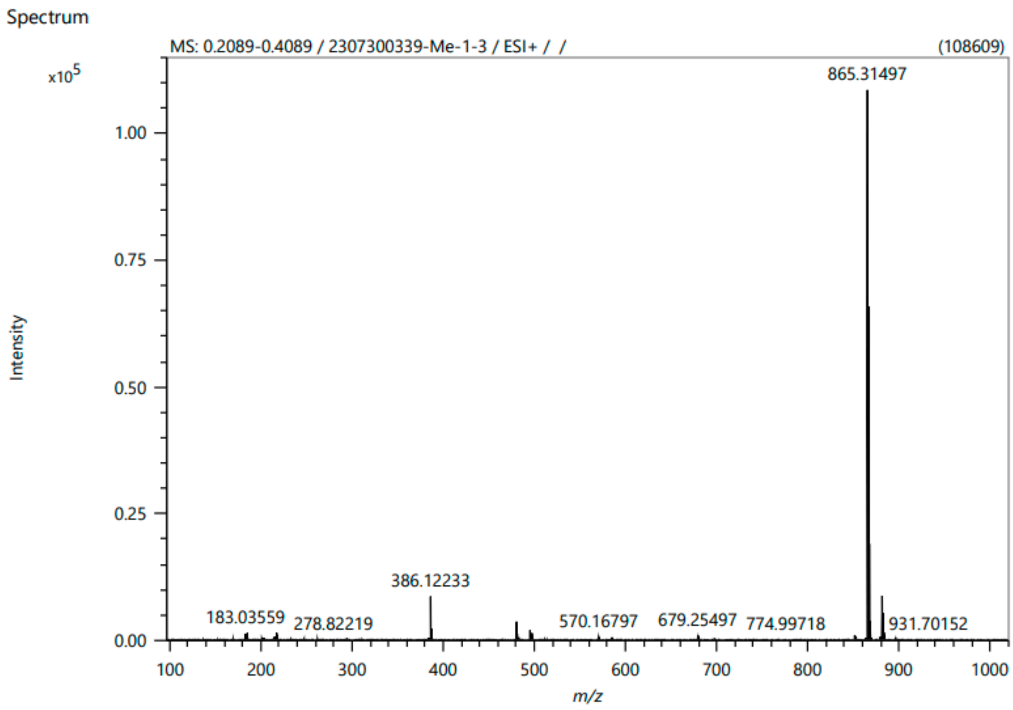

Figure S20. High Resolution Mass Spectrometry of **L5**

3. GC-MS spectrum of the typical oligomerization products

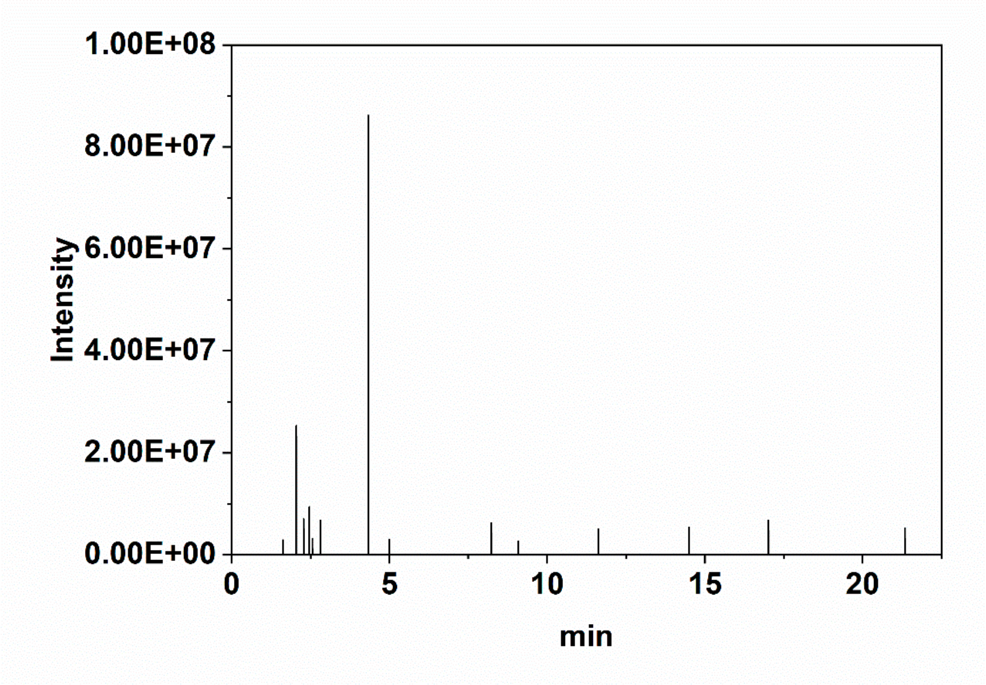

Figure S21. GC-MS spectrum of the oligomerization product obtained from entry 2 of Table 1.

Table S2. Corresponding residence time of chromatographic peak to the product.

Residence time	Oligomerization product
1.632	1-Butene
2.054	1-Hexene
2.287	Cyclopentane, methyl-
2.459	Cyclopentane, methylene-
2.562	Cyclohexane
2.822	Cyclopentane, 1,2-dimethy-
4.337	1-Octene
4.998	Cyclopentane, propyl-
8.241	1-Decene
9.086	Cyclopentane, pentyl-
11.626	1-Dodecene
14.496	1-Tetradecene
17.021	7-Hexadecene, (Z)-
21.333	1-Octadecene

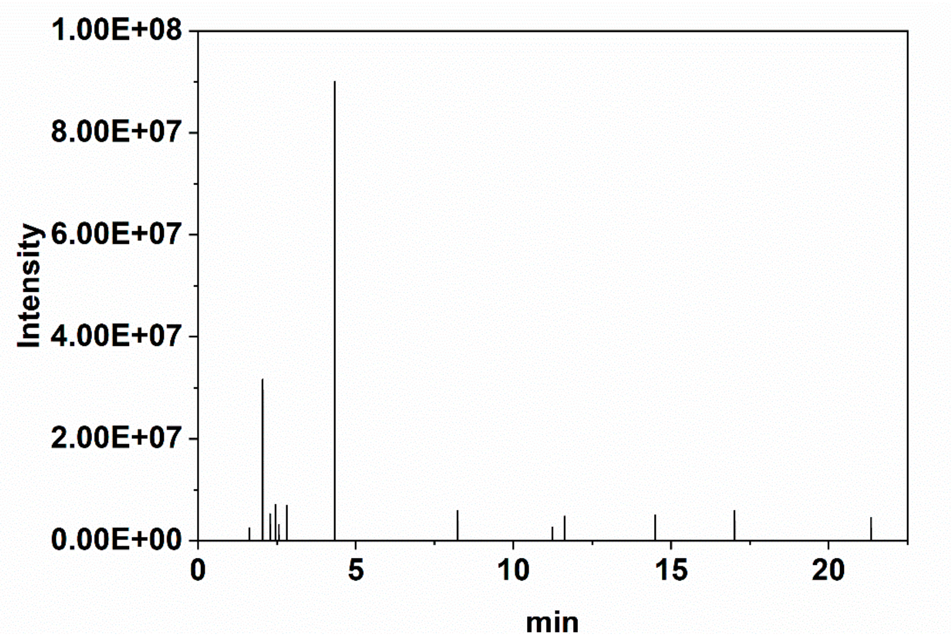

Figure S22. GC-MS spectrum of the oligomerization product obtained from entry 5 of Table 1.

Table S3. Corresponding residence time of chromatographic peak to the product.

Residence time	Liquid substance
1.632	1-Butene
2.054	1-Hexene
2.287	Cyclopentane, methyl-
2.459	Cyclopentane, methylene-
2.562	Cyclohexane
2.822	Cyclopentane, 1,2-dimethy-
4.337	1-Octene
4.998	Cyclopentane, propyl-
8.241	1-Decene
11.231	Decane, 5-methyl-6-methylene-1-
11.626	Dodecene
14.496	1-Tetradecene
17.021	7-Hexadecene, (Z)-
21.333	1-Octadecene

Computational methodology

All the density functional theory (DFT) calculations were performed by Gaussian 09 program [4] packages with M062X functional. Geometry optimizations were carried out in the gas phase with the 6-31G (d,p) basis set.

Optimized geometries from DFT calculations

L1

**C**	**0.58008**	**1.23631**	**-0.38497**
**C**	**-0.78153**	**1.18921**	**-0.07028**
**C**	**-1.38427**	**0.00851**	**0.40126**
**C**	**-0.53437**	**-1.10524**	**0.55055**
**C**	**0.80359**	**-1.06179**	**0.19658**
**C**	**1.41361**	**0.10672**	**-0.27646**
**N**	**2.80371**	**0.1548**	**-0.59401**
**N**	**-2.77732**	**-0.07698**	**0.69605**
**P**	**3.78463**	**-1.19751**	**-1.21556**
**C**	**4.23573**	**-2.34493**	**0.18315**
**C**	**5.38195**	**-3.11108**	**-0.11244**
**C**	**5.91249**	**-4.01589**	**0.81066**
**C**	**5.32811**	**-4.15198**	**2.0717**
**C**	**4.21504**	**-3.37442**	**2.3955**
**C**	**3.67244**	**-2.48333**	**1.46331**
**C**	**2.65081**	**-2.18842**	**-2.30811**
**C**	**2.64405**	**-1.76238**	**-3.65329**
**C**	**1.87097**	**-2.41164**	**-4.61633**
**C**	**1.10925**	**-3.52529**	**-4.25676**
**C**	**1.1346**	**-3.9877**	**-2.9345**
**C**	**1.89442**	**-3.32587**	**-1.96855**

P	-4.0144	1.16592	0.53
C	-3.48448	2.6741	1.48185
C	-3.03471	3.88202	0.92852
C	-2.71015	4.96458	1.7481
C	-2.82324	4.85462	3.13579
C	-3.28469	3.66244	3.69931
C	-3.62519	2.58751	2.87829
C	-3.98447	1.70363	-1.23154
C	-4.88046	2.72732	-1.60086
C	-5.03977	3.08955	-2.93765
C	-4.32125	2.42422	-3.93526
C	-3.44089	1.39924	-3.58155
C	-3.27119	1.04168	-2.24202
P	-3.68986	-1.43796	1.45892
C	-4.08569	-2.52869	0.01451
C	-5.32629	-3.18514	0.0542
C	-5.72759	-4.02656	-0.98731
C	-4.89893	-4.21083	-2.09365
C	-3.66924	-3.54886	-2.15558
C	-3.26483	-2.71308	-1.11132
C	-2.99456	-1.92266	3.46172
C	-2.45149	-0.87085	4.20541
C	-1.27031	-1.03732	4.93867
C	-0.64133	-2.27745	4.95595
C	-1.19717	-3.34995	4.24402
C	-2.32659	-3.1614	3.45308
P	3.88541	1.54573	-0.48639
C	3.17557	2.90949	-1.53847
C	3.15428	2.69055	-2.92723
C	2.76479	3.70343	-3.80504

C	2.41431	4.96518	-3.31266
C	2.44679	5.19921	-1.93755
C	2.81891	4.17877	-1.05542
C	3.72921	2.19432	1.23788
C	2.83855	1.7294	2.22046
C	2.88857	2.23572	3.52148
C	3.82197	3.21481	3.86464
C	4.72322	3.67782	2.89923
C	4.68423	3.16326	1.60389
H	0.98102	2.1838	-0.72852
H	-1.352	2.10174	-0.19011
H	-0.94838	-2.01479	1.04901
H	1.38816	-1.95518	0.34798
H	5.86589	-2.98987	-1.07925
H	6.79184	-4.60054	0.55011
H	5.7475	-4.84427	2.79997
H	3.76268	-3.45608	3.38067
H	2.82308	-1.87938	1.75996
H	3.26019	-0.91363	-3.94682
H	1.87734	-2.05781	-5.64798
H	0.51257	-4.0416	-5.00396
H	0.56258	-4.87094	-2.65658
H	1.92851	-3.7275	-0.95456
H	-2.95417	3.98553	-0.15009
H	-2.36432	5.89268	1.30058
H	-2.56342	5.69599	3.77246
H	-3.39022	3.57379	4.77742
H	-4.00856	1.6726	3.32239
H	-5.4648	3.2402	-0.83894
H	-5.73409	3.88473	-3.20023

H	-4.45142	2.69979	-4.97834
H	-2.87823	0.87392	-4.34946
H	-2.58544	0.24118	-1.98367
H	-5.98602	-3.02693	0.90375
H	-6.69169	-4.52716	-0.93925
H	-5.21211	-4.85892	-2.90919
H	-3.02276	-3.68162	-3.01887
H	-2.3108	-2.19819	-1.17644
H	-2.95149	0.09601	4.20515
H	-0.85899	-0.20184	5.49802
H	0.26383	-2.42542	5.53987
H	-0.73402	-4.33295	4.30559
H	-2.78552	-4.02016	2.97295
H	3.44699	1.71969	-3.32169
H	2.74626	3.5134	-4.87461
H	2.12458	5.76159	-3.99636
H	2.17774	6.17818	-1.54632
H	2.83443	4.374	0.01209
H	2.10427	0.96971	1.97852
H	2.18801	1.86246	4.26731
H	3.85407	3.60766	4.87669
H	5.46552	4.42982	3.15692
H	5.40881	3.51371	0.86907

L2

C	-0.41949	0.41795	-0.3509
C	1.03167	0.15542	-0.7564
C	2.01761	0.50164	0.36758
C	1.63155	-0.14104	1.70913
C	0.17832	0.19029	2.06504

C	-0.73829	-0.32012	0.95218
N	-2.1794	-0.30943	1.33213
N	3.45054	0.29658	-0.01232
P	-2.33926	-1.05717	2.93066
C	-1.40557	-0.54292	4.42854
C	-2.15421	-0.25508	5.57214
C	-1.51996	0.1172	6.75495
C	-0.13301	0.21753	6.79644
C	0.62235	-0.08399	5.66333
C	-0.0104	-0.48203	4.49139
C	-1.32322	-2.59055	2.69169
C	-1.73753	-3.72473	3.40075
C	-1.04149	-4.92431	3.2945
C	0.07669	-5.01192	2.46563
C	0.48589	-3.89495	1.74408
C	-0.2147	-2.69472	1.85048
P	-3.25264	-0.43682	-0.02266
C	-3.04841	1.15987	-0.9227
C	-3.51639	2.29473	-0.24696
C	-3.45181	3.55245	-0.83662
C	-2.94652	3.68973	-2.12751
C	-2.49467	2.56533	-2.81252
C	-2.53516	1.30916	-2.21342
C	-2.48746	-1.67591	-1.14598
C	-3.05711	-1.87071	-2.41184
C	-2.60353	-2.88614	-3.24706
C	-1.59337	-3.74514	-2.81937
C	-1.05042	-3.58698	-1.54771
C	-1.50048	-2.56409	-0.71662
P	4.43296	0.9166	1.33199

C	4.13315	-0.22047	2.7506
C	3.23108	0.0137	3.79466
C	3.08501	-0.91343	4.81987
C	3.83145	-2.09047	4.81414
C	4.74087	-2.32884	3.78854
C	4.90245	-1.38997	2.77322
C	3.48999	2.42048	1.82274
C	2.52939	3.00428	0.99521
C	1.93405	4.21533	1.34298
C	2.29781	4.86171	2.51984
C	3.27082	4.2974	3.34336
C	3.87088	3.09394	2.99083
P	3.94521	0.7902	-1.66186
C	4.3618	-0.71438	-2.63582
C	5.48116	-0.63605	-3.46747
C	5.88387	-1.74039	-4.215
C	5.17847	-2.93709	-4.12531
C	4.06289	-3.02441	-3.29437
C	3.65588	-1.91797	-2.55563
C	2.90344	1.36134	-3.08349
C	2.97226	2.71879	-3.41986
C	2.14818	3.25607	-4.40677
C	1.27978	2.42115	-5.10534
C	1.24162	1.0579	-4.81871
C	2.04271	0.53249	-3.81024
H	-1.09783	0.08099	-1.14346
H	-0.58779	1.4934	-0.2002
H	1.13199	-0.90254	-1.02117
H	1.30038	0.73346	-1.64877
H	1.91613	1.58558	0.53603

**H**	**1.72839**	**-1.22717**	**1.67651**
**H**	**2.31247**	**0.21894**	**2.48781**
**H**	**-0.09115**	**-0.27538**	**3.01996**
**H**	**0.04139**	**1.27292**	**2.18469**
**H**	**-0.47199**	**-1.3778**	**0.79847**
**H**	**-3.23942**	**-0.29397**	**5.52523**
**H**	**-2.11185**	**0.34811**	**7.6353**
**H**	**0.36295**	**0.52133**	**7.71337**
**H**	**1.70572**	**-0.02116**	**5.69635**
**H**	**0.58097**	**-0.75336**	**3.62365**
**H**	**-2.61167**	**-3.66456**	**4.04613**
**H**	**-1.37173**	**-5.79233**	**3.85666**
**H**	**0.62032**	**-5.94789**	**2.38142**
**H**	**1.3487**	**-3.95492**	**1.08734**
**H**	**0.09356**	**-1.83516**	**1.26363**
**H**	**-3.93051**	**2.19008**	**0.75385**
**H**	**-3.80875**	**4.42229**	**-0.29394**
**H**	**-2.90621**	**4.66786**	**-2.59665**
**H**	**-2.08177**	**2.66668**	**-3.81172**
**H**	**-2.12311**	**0.454**	**-2.73952**
**H**	**-3.8622**	**-1.2211**	**-2.74833**
**H**	**-3.04579**	**-3.01283**	**-4.23029**
**H**	**-1.24276**	**-4.54087**	**-3.46884**
**H**	**-0.27524**	**-4.26248**	**-1.19878**
**H**	**-1.08166**	**-2.4407**	**0.27669**
**H**	**2.61722**	**0.90853**	**3.79025**
**H**	**2.36902**	**-0.72613**	**5.61414**
**H**	**3.70695**	**-2.81728**	**5.61107**
**H**	**5.33237**	**-3.23882**	**3.78233**
**H**	**5.62354**	**-1.57088**	**1.97936**

H	2.24739	2.49359	0.07857
H	1.18469	4.65337	0.69067
H	1.83354	5.80446	2.79134
H	3.56521	4.79838	4.26032
H	4.63539	2.6657	3.63586
H	6.05727	0.28547	-3.50638
H	6.76003	-1.67123	-4.85241
H	5.49937	-3.80239	-4.69722
H	3.51426	-3.95812	-3.21217
H	2.80694	-1.98937	-1.88404
H	3.66647	3.36631	-2.88963
H	2.17419	4.32003	-4.62873
H	0.64103	2.83551	-5.8791
H	0.5784	0.40368	-5.37644
H	1.99189	-0.52706	-3.58231

L3

C	-0.8463	0.94181	-4.99823
C	0.52954	0.34186	-5.33731
C	1.48881	0.44133	-4.13818
C	0.87551	-0.27908	-2.92853
C	-0.50617	0.30219	-2.57646
C	-1.47374	0.25508	-3.77505
N	-2.85877	0.7321	-3.45218
N	1.93781	-0.73435	-1.32861
P	-4.18763	-0.36699	-3.10296
C	-3.8443	-1.11251	-1.43728
C	-3.21645	-0.36946	-0.42555
C	-3.13768	-0.85569	0.88041
C	-3.70012	-2.08982	1.20686

C	-4.3501	-2.82945	0.21735
C	-4.42715	-2.3438	-1.08776
C	-3.89436	-1.80327	-4.24415
C	-4.62656	-1.79871	-5.44372
C	-4.49708	-2.8341	-6.37038
C	-3.6435	-3.90536	-6.10348
C	-2.91787	-3.93254	-4.91056
C	-3.03911	-2.88944	-3.99109
P	-3.47437	2.3729	-3.37388
C	-2.1274	3.4211	-2.63325
C	-2.16331	3.55347	-1.23378
C	-1.23428	4.34575	-0.55888
C	-0.26109	5.0442	-1.27628
C	-0.22589	4.94481	-2.66916
C	-1.15073	4.14217	-3.33963
C	-3.59187	2.99464	-5.11681
C	-3.6991	4.37583	-5.36019
C	-3.99399	4.85903	-6.63497
C	-4.20604	3.97298	-7.69249
C	-4.13074	2.5995	-7.45954
C	-3.83481	2.11628	-6.18433
P	2.24242	-2.28997	-0.57362
C	1.82962	-3.58888	-1.83756
C	0.57413	-4.18719	-2.03674
C	0.39779	-5.16942	-3.01104
C	1.47403	-5.57423	-3.80443
C	2.73007	-4.99946	-3.6109
C	2.90528	-4.02247	-2.62952
C	0.94607	-2.56051	0.7277
C	0.44292	-1.48616	1.47877

C	-0.36795	-1.70378	2.59303
C	-0.67957	-3.00266	2.99602
C	-0.16101	-4.08232	2.27991
C	0.64699	-3.8646	1.1638
P	3.15482	0.41617	-0.79646
C	3.73525	1.1944	-2.37597
C	4.102	2.54872	-2.45253
C	4.698	3.06584	-3.60461
C	4.94533	2.24018	-4.7017
C	4.60794	0.88716	-4.63131
C	4.02017	0.36942	-3.47789
C	2.20078	1.80101	0.00621
C	2.29768	1.86272	1.40704
C	1.60262	2.82838	2.13807
C	0.79862	3.75646	1.47675
C	0.70031	3.71861	0.08388
C	1.39767	2.75448	-0.64417
H	-1.50478	0.83874	-5.86374
H	-0.73593	2.01344	-4.81144
H	0.39926	-0.70042	-5.63273
H	0.95106	0.86596	-6.19918
H	1.6505	1.47084	-3.89555
H	0.78735	-1.35133	-3.14319
H	-0.92904	-0.25885	-1.73953
H	-0.38879	1.33773	-2.23883
H	-1.59053	-0.79943	-4.04716
H	-2.78155	0.59527	-0.66296
H	-2.63615	-0.26675	1.64345
H	-3.63901	-2.46884	2.22277
H	-4.79848	-3.78881	0.46042

H	-4.94383	-2.93216	-1.84044
H	-5.31393	-0.98058	-5.64293
H	-5.07358	-2.81196	-7.29069
H	-3.55283	-4.72081	-6.81551
H	-2.26008	-4.76936	-4.69187
H	-2.48	-2.93046	-3.06163
H	-2.93902	3.04034	-0.67116
H	-1.28286	4.43268	0.52284
H	0.45366	5.67582	-0.75635
H	0.52266	5.48966	-3.23754
H	-1.11909	4.09394	-4.4226
H	-3.55052	5.08347	-4.54956
H	-4.05915	5.93087	-6.80058
H	-4.43599	4.34936	-8.68494
H	-4.30373	1.89904	-8.27203
H	-3.79004	1.04555	-6.01611
H	-0.27257	-3.88004	-1.43127
H	-0.58472	-5.60732	-3.16161
H	1.33411	-6.33813	-4.5639
H	3.57512	-5.31744	-4.21488
H	3.89098	-3.59352	-2.46755
H	0.68887	-0.4692	1.19485
H	-0.75368	-0.85306	3.14814
H	-1.31065	-3.17208	3.86358
H	-0.38483	-5.09971	2.58906
H	1.04706	-4.72088	0.62954
H	3.9215	3.20777	-1.60958
H	4.96932	4.11743	-3.6421
H	5.40544	2.64396	-5.59885
H	4.80723	0.23156	-5.47444

**H**	**3.77994**	**-0.68797**	**-3.42769**
**H**	**2.92673**	**1.1445**	**1.92638**
**H**	**1.6943**	**2.85721**	**3.22009**
**H**	**0.25623**	**4.50958**	**2.04134**
**H**	**0.07828**	**4.43749**	**-0.44157**
**H**	**1.32232**	**2.76103**	**-1.72661**
**H**	**2.42262**	**-0.01644**	**-4.38984**

L4

**C**	**-0.12724**	**2.54547**	**1.02098**
**C**	**0.5321**	**1.6655**	**2.09085**
**C**	**0.97168**	**0.31352**	**1.51936**
**C**	**1.92043**	**0.53207**	**0.32626**
**C**	**1.17089**	**1.27865**	**-0.78791**
**C**	**0.77997**	**2.66056**	**-0.21303**
**N**	**2.25809**	**2.46246**	**3.19188**
**N**	**-0.46574**	**-1.5767**	**1.11295**
**P**	**3.50447**	**1.43305**	**3.89199**
**C**	**4.91436**	**1.48008**	**2.69499**
**C**	**5.0951**	**2.52335**	**1.77742**
**C**	**6.27104**	**2.61311**	**1.02852**
**C**	**7.28379**	**1.66644**	**1.18597**
**C**	**7.11408**	**0.62063**	**2.09782**
**C**	**5.94378**	**0.53404**	**2.84856**
**C**	**2.8067**	**-0.25847**	**3.57146**
**C**	**1.89796**	**-0.76286**	**4.51605**
**C**	**1.23056**	**-1.96828**	**4.29388**
**C**	**1.47847**	**-2.70151**	**3.13113**
**C**	**2.40322**	**-2.22786**	**2.19749**
**C**	**3.05893**	**-1.01544**	**2.41464**

P	2.25501	4.00381	4.00702
C	1.88282	5.2835	2.71718
C	2.95402	6.10857	2.34016
C	2.77519	7.13905	1.41282
C	1.52415	7.34674	0.83328
C	0.44532	6.5385	1.20253
C	0.6224	5.52881	2.14526
C	0.71474	4.03515	5.0391
C	0.00847	5.22213	5.29311
C	-1.04659	5.24498	6.20738
C	-1.40909	4.08666	6.89479
C	-0.69743	2.90561	6.6715
C	0.35769	2.88474	5.76188
P	-0.62299	-2.76116	-0.17225
C	0.97158	-3.71162	-0.05081
C	2.20248	-3.32146	-0.60452
C	3.35614	-4.07052	-0.37508
C	3.30053	-5.22445	0.40973
C	2.08165	-5.63346	0.95382
C	0.92711	-4.88558	0.71844
C	-0.32667	-1.74714	-1.69457
C	-0.69263	-0.39314	-1.7353
C	-0.58967	0.34279	-2.91661
C	-0.1189	-0.26346	-4.08205
C	0.23266	-1.61595	-4.06194
C	0.1235	-2.35196	-2.88163
P	-2.00061	-1.34344	1.89726
C	-1.63687	-1.17383	3.69838
C	-2.36126	-0.31281	4.53989
C	-2.20885	-0.37092	5.92681

**C**	**-1.32825**	**-1.28524**	**6.50416**
**C**	**-0.62221**	**-2.16706**	**5.68102**
**C**	**-0.79047**	**-2.12387**	**4.29903**
**C**	**-2.65235**	**0.32757**	**1.39974**
**C**	**-3.63554**	**0.27354**	**0.39614**
**C**	**-4.26282**	**1.43091**	**-0.067**
**C**	**-3.92741**	**2.66688**	**0.48406**
**C**	**-2.9473**	**2.7399**	**1.47743**
**C**	**-2.3112**	**1.58389**	**1.92861**
**H**	**-0.31599**	**3.54397**	**1.42107**
**H**	**-1.10257**	**2.13003**	**0.7521**
**H**	**-0.15781**	**1.51607**	**2.92908**
**H**	**2.76267**	**1.1121**	**0.64106**
**H**	**1.81404**	**1.42306**	**-1.65641**
**H**	**0.29275**	**0.71425**	**-1.10579**
**H**	**1.6709**	**3.18639**	**0.0602**
**H**	**4.31982**	**3.27159**	**1.64832**
**H**	**6.38529**	**3.41928**	**0.31346**
**H**	**8.19633**	**1.7401**	**0.60158**
**H**	**7.89379**	**-0.12514**	**2.22532**
**H**	**5.82548**	**-0.28148**	**3.55789**
**H**	**1.70895**	**-0.20679**	**5.4307**
**H**	**0.52278**	**-2.33743**	**5.03055**
**H**	**0.96087**	**-3.64111**	**2.95913**
**H**	**2.61336**	**-2.78683**	**1.29023**
**H**	**3.74911**	**-0.65043**	**1.66414**
**H**	**3.93106**	**5.94755**	**2.78791**
**H**	**3.61287**	**7.77555**	**1.14273**
**H**	**1.38586**	**8.13288**	**0.09713**
**H**	**-0.52665**	**6.68555**	**0.74351**

H	-0.22992	4.93112	2.44494
H	0.27417	6.1359	4.77125
H	-1.58752	6.17164	6.37836
H	-2.23256	4.10442	7.60267
H	-0.96378	1.99901	7.20787
H	0.90611	1.96324	5.59845
H	2.26926	-2.42409	-1.21097
H	4.30025	-3.74429	-0.80267
H	4.20084	-5.80436	0.59144
H	2.02904	-6.53553	1.55669
H	-0.02042	-5.20979	1.14181
H	-1.06098	0.09472	-0.84098
H	-0.86391	1.39156	-2.9172
H	-0.02795	0.3134	-4.99775
H	0.59281	-2.09976	-4.96588
H	0.40662	-3.40101	-2.88105
H	-3.05201	0.40827	4.11824
H	-2.77785	0.30903	6.55526
H	-1.20512	-1.32391	7.58254
H	0.05157	-2.89906	6.11742
H	-0.26138	-2.83606	3.67472
H	-3.91463	-0.69128	-0.01906
H	-5.01923	1.36363	-0.84338
H	-4.42488	3.57076	0.14298
H	-2.6717	3.69863	1.90291
H	-1.56217	1.66757	2.70614
H	1.46737	-0.28131	2.28981
H	0.26694	3.21741	-0.96908
H	2.25615	-0.41501	-0.04147

L5

C	-1.22819	0.94711	2.11411
C	-0.10511	0.3912	2.99918
C	0.44683	-0.9287	2.45063
C	0.9341	-0.73205	1.00313
C	-0.2552	-0.32944	0.11768
C	-0.76775	1.02913	0.65112
N	-1.71101	1.75633	-0.24106
N	1.8136	-1.83589	0.51418
P	-1.22704	3.23526	-1.06679
C	-0.34093	2.68163	-2.5936
C	-0.5339	1.41724	-3.16552
C	0.00806	1.11507	-4.41721
C	0.7469	2.06949	-5.11708
C	0.94671	3.33404	-4.55584
C	0.40181	3.63727	-3.31011
C	0.18845	3.8021	-0.00568
C	-0.13062	4.5706	1.12572
C	0.85464	4.93304	2.04535
C	2.18176	4.55084	1.83622
C	2.51919	3.81318	0.69904
C	1.53043	3.43961	-0.21194
P	-3.40635	1.46339	-0.52433
C	-3.60663	-0.37373	-0.68136
C	-3.87447	-0.85663	-1.97187
C	-4.09264	-2.21861	-2.19827
C	-4.02625	-3.1215	-1.13786
C	-3.7672	-2.65653	0.1545
C	-3.57489	-1.29563	0.37931
C	-4.26244	1.81001	1.08365

C	-5.41113	1.1093	1.48583
C	-6.13033	1.50335	2.61589
C	-5.72407	2.61157	3.35918
C	-4.5988	3.33357	2.9546
C	-3.88407	2.9419	1.82461
P	3.5498	-1.59437	0.596
C	3.78065	-0.84915	2.28482
C	3.60887	0.50609	2.6131
C	3.72308	0.93911	3.93375
C	4.01348	0.02635	4.95017
C	4.20233	-1.32105	4.63737
C	4.09316	-1.75156	3.31434
C	3.80361	-0.11573	-0.49153
C	2.96247	0.11781	-1.59009
C	3.22334	1.15586	-2.4857
C	4.33348	1.98068	-2.29953
C	5.19072	1.74962	-1.22019
C	4.93196	0.70767	-0.32903
P	1.45928	-3.33045	-0.30198
C	0.03986	-4.08627	0.60306
C	-0.97338	-4.81122	-0.04657
C	-1.90567	-5.55124	0.68374
C	-1.85055	-5.58101	2.07689
C	-0.83108	-4.88756	2.73526
C	0.11185	-4.16713	2.00593
C	0.76847	-2.919	-1.98068
C	1.70413	-3.04658	-3.02235
C	1.34495	-2.81752	-4.35127
C	0.03027	-2.47341	-4.66268
C	-0.91109	-2.33529	-3.63921

**C**	**-0.54673**	**-2.55036**	**-2.31048**
**H**	**-2.1179**	**0.31827**	**2.20954**
**H**	**0.71049**	**1.12474**	**3.05433**
**H**	**-0.4701**	**0.25265**	**4.02315**
**H**	**-0.33984**	**-1.6899**	**2.46541**
**H**	**1.58898**	**0.14475**	**1.00607**
**H**	**0.05871**	**-0.20942**	**-0.91956**
**H**	**-1.03474**	**-1.09232**	**0.15038**
**H**	**0.12065**	**1.66023**	**0.67575**
**H**	**-1.11204**	**0.66619**	**-2.63707**
**H**	**-0.14014**	**0.12655**	**-4.83571**
**H**	**1.16646**	**1.83017**	**-6.08989**
**H**	**1.52447**	**4.08362**	**-5.0894**
**H**	**0.56032**	**4.62586**	**-2.88611**
**H**	**-1.15807**	**4.88512**	**1.28986**
**H**	**0.58741**	**5.51833**	**2.92053**
**H**	**2.94931**	**4.83497**	**2.55071**
**H**	**3.54658**	**3.51173**	**0.5165**
**H**	**1.8076**	**2.84149**	**-1.07117**
**H**	**-3.92413**	**-0.15587**	**-2.80103**
**H**	**-4.31116**	**-2.57229**	**-3.20173**
**H**	**-4.17467**	**-4.18283**	**-1.3128**
**H**	**-3.69913**	**-3.35621**	**0.98085**
**H**	**-3.40648**	**-0.9464**	**1.39082**
**H**	**-5.74692**	**0.24506**	**0.9217**
**H**	**-7.00855**	**0.93841**	**2.91608**
**H**	**-6.28171**	**2.91483**	**4.24041**
**H**	**-4.27778**	**4.20441**	**3.51953**
**H**	**-3.01373**	**3.51156**	**1.517**
**H**	**3.37519**	**1.23057**	**1.83976**

H	3.57436	1.98974	4.16754
H	4.09755	0.3643	5.97905
H	4.43858	-2.03481	5.42139
H	4.24408	-2.80143	3.07523
H	2.09627	-0.51234	-1.75167
H	2.5488	1.32487	-3.3173
H	4.52873	2.79559	-2.99043
H	6.06241	2.38102	-1.07062
H	5.60473	0.54497	0.50857
H	-1.04049	-4.80518	-1.1284
H	-2.68207	-6.09994	0.15727
H	-2.58103	-6.1508	2.64372
H	-0.76253	-4.91847	3.81905
H	0.91691	-3.66095	2.52801
H	2.72409	-3.3366	-2.78418
H	2.08573	-2.92211	-5.13853
H	-0.26251	-2.31232	-5.69668
H	-1.93297	-2.05606	-3.8715
H	-1.29814	-2.44605	-1.53776
H	1.26614	-1.28747	3.07765
C	-1.63379	2.35262	2.59539
H	-1.94176	2.94167	1.7569
H	-2.44288	2.27176	3.29091
H	-0.79854	2.82098	3.07278

Reference

1. Dolomanov, O.V., Bourhis, L.J., Gildea, R.J, Howard, J.A.K. & Puschmann, H. (2009), J. Appl. Cryst. 42, 339-341.

2. Sheldrick, G.M. (2015). Acta Cryst. A71, 3-8.

3. Sheldrick, G.M. (2015). Acta Cryst. C71, 3-8.

4. Gaussian 09, Revision E.01, M. J. Frisch, G. W. Trucks, H. B. Schlegel, G. E. Scuseria, M. A. Robb, J. R. Cheeseman, G. Scalmani, V. Barone, B. Mennucci, G. A. Petersson, H. Nakatsuji, M. Caricato, X. Li, H. P. Hratchian, A. F. Izmaylov, J. Bloino, G. Zheng, J. L. Sonnenberg, M. Hada, M. Ehara, K. Toyota, R. Fukuda, J. Hasegawa, M. Ishida, T. Nakajima, Y. Honda, O. Kitao, H. Nakai, T. Vreven, J. A. Montgomery, Jr., J. E. Peralta, F. Ogliaro, M. Bearpark, J. J. Heyd, E. Brothers, K. N. Kudin, V. N. Staroverov, T. Keith, R. Kobayashi, J. Normand, K. Raghavachari, A. Rendell, J. C. Burant, S. S. Iyengar, J. Tomasi, M. Cossi, N. Rega, J. M. Millam, M. Klene, J. E. Knox, J. B. Cross, V. Bakken, C. Adamo, J. Jaramillo, R. Gomperts, R. E. Stratmann, O. Yazyev, A. J. Austin, R. Cammi, C. Pomelli, J. W. Ochterski, R. L. Martin, K. Morokuma, V. G. Zakrzewski, G. A. Voth, P. Salvador, J. J. Dannenberg, S. Dapprich, A. D. Daniels, O. Farkas, J. B. Foresman, J. V. Ortiz, J. Cioslowski, and D. J. Fox, Gaussian, Inc., Wallingford CT, **2013**.
